# Consensus guidelines for sarcopenia prevention, diagnosis and management in Australia and New Zealand

**DOI:** 10.1002/jcsm.13115

**Published:** 2022-11-09

**Authors:** Jesse Zanker, Marc Sim, Kate Anderson, Saliu Balogun, Sharon L. Brennan‐Olsen, Elsa Dent, Gustavo Duque, Christian M. Girgis, Mathis Grossmann, Alan Hayes, Tim Henwood, Vasant Hirani, Charles Inderjeeth, Sandra Iuliano, Justin Keogh, Joshua R. Lewis, Gordon S. Lynch, Julie A. Pasco, Steven Phu, Esmee M. Reijnierse, Nicholas Russell, Lara Vlietstra, Renuka Visvanathan, Troy Walker, Debra L. Waters, Solomon Yu, Andrea B. Maier, Robin M. Daly, David Scott

**Affiliations:** ^1^ Australian Institute for Musculoskeletal Science (AIMSS) The University of Melbourne and Western Health St. Albans Victoria Australia; ^2^ Department of Medicine ‐ Western Health The University of Melbourne St. Albans Victoria Australia; ^3^ Nutrition & Health Innovation Research Institute, School of Medical and Health Sciences Edith Cowan University Joondalup Western Australia Australia; ^4^ School of Medicine University of Western Australia Perth Western Australia Australia; ^5^ Institute for Health Transformation – Determinants of Health, Faculty of Health Deakin University Burwood Victoria Australia; ^6^ School of Health and Social Development, Faculty of Health Deakin University Burwood Victoria Australia; ^7^ College of Health and Medicine Australian National University Canberra Australian Capital Territory Australia; ^8^ Menzies Institute for Medical Research University of Tasmania Hobart Tasmania Australia; ^9^ School of Health and Social Development, Faculty of Health Deakin University Geelong Victoria Australia; ^10^ Institute for Health Transformation Deakin University Geelong Victoria Australia; ^11^ Torrens University Australia Adelaide South Australia Australia; ^12^ Research Institute of the McGill University Health Centre Department of Medicine, McGill University Montreal Quebec Canada; ^13^ Faculty of Medicine and Health University of Sydney Sydney New South Wales Australia; ^14^ Department of Diabetes and Endocrinology Westmead Hospital Westmead New South Wales Australia; ^15^ Department of Medicine ‐ Austin Health, Department of Endocrinology The University of Melbourne Melbourne Victoria Australia; ^16^ Institute for Health and Sport (IHeS) Victoria University Footscray Victoria Australia; ^17^ Human Movement and Nutritional Science University of Queensland Brisbane Queensland Australia; ^18^ Nutrition and Dietetics Group, School of Life and Environmental Sciences Charles Perkins Centre University of Sydney New South Wales Sydney Australia; ^19^ North Metropolitan Health Service & University of Western Australia Perth Western Australia Australia; ^20^ Faculty of Health Sciences and Medicine Bond University Gold Coast Queensland Australia; ^21^ Human Potential Centre Auckland University of Technology Auckland New Zealand; ^22^ Cluster for Health Improvement, Faculty of Science, Health, Education and Engineering University of the Sunshine Coast Sunshine Coast Queensland Australia; ^23^ Kasturba Medical College, Mangalore Manipal Academy of Higher Education Manipal India; ^24^ Centre for Kidney Research, Children's Hospital at Westmead School of Public Health, Sydney Medical School The University of Sydney Sydney New South Wales Australia; ^25^ Centre for Muscle Research, Department of Anatomy and Physiology, School of Biomedical Sciences The University of Melbourne Melbourne Victoria Australia; ^26^ IMPACT‐Institute for Mental and Physical Health and Clinical Translation, Barwon Health Deakin University Geelong Victoria Australia; ^27^ Falls, Balance, and Injury Research Centre Neuroscience Research Australia (NeuRA) Sydney New South Wales Australia; ^28^ Department of Medicine and Aged Care, @AgeMelbourne, The Royal Melbourne Hospital The University of Melbourne Parkville Victoria Australia; ^29^ Amsterdam UMC location Vrije Universiteit Amsterdam, Rehabilitation Medicine Amsterdam The Netherlands; ^30^ Amsterdam Movement Sciences, Ageing & Vitality Amsterdam The Netherlands; ^31^ School of Physical Education, Sport and Exercise Sciences University of Otago Dunedin New Zealand; ^32^ Adelaide Geriatrics Training and Research with Aged Care (GTRAC) Centre, School of Medicine, Faculty of Health and Medical Sciences University of Adelaide Adelaide South Australia Australia; ^33^ Aged & Extended Care Services, Acute and Urgent Care The Queen Elizabeth Hospital, Central Adelaide Local Health Network Adelaide South Australia Australia; ^34^ Institute for Health Transformation, Global Obesity Centre Deakin University Geelong Victoria Australia; ^35^ Healthy Longevity Translational Research Program, Yong Loo Lin School of Medicine National University of Singapore Singapore; ^36^ Centre for Healthy Longevity, @AgeSingapore National University Health System Singapore; ^37^ Department of Human Movement Sciences, @AgeAmsterdam, Faculty of Behavioural and Movement Sciences Vrije Universiteit Amsterdam Amsterdam The Netherlands; ^38^ Institute for Physical Activity and Nutrition Deakin University Burwood Victoria Australia; ^39^ Department of Medicine, School of Clinical Sciences at Monash Health Monash University Clayton Victoria Australia

**Keywords:** Aged, Mass screening, Geriatric assessment, Sarcopenia

## Abstract

**Background:**

Sarcopenia is an age‐associated skeletal muscle condition characterized by low muscle mass, strength, and physical performance. There is no international consensus on a sarcopenia definition and no contemporaneous clinical and research guidelines specific to Australia and New Zealand. The Australian and New Zealand Society for Sarcopenia and Frailty Research (ANZSSFR) Sarcopenia Diagnosis and Management Task Force aimed to develop consensus guidelines for sarcopenia prevention, assessment, management and research, informed by evidence, consumer opinion, and expert consensus, for use by health professionals and researchers in Australia and New Zealand.

**Methods:**

A four‐phase modified Delphi process involving topic experts and informed by consumers, was undertaken between July 2020 and August 2021. Phase 1 involved a structured meeting of 29 Task Force members and a systematic literature search from which the Phase 2 online survey was developed (Qualtrics). Topic experts responded to 18 statements, using 11‐point Likert scales with agreement threshold set *a priori* at >80%, and five multiple‐choice questions. Statements with moderate agreement (70%–80%) were revised and re‐introduced in Phase 3, and statements with low agreement (<70%) were rejected. In Phase 3, topic experts responded to six revised statements and three additional questions, incorporating results from a parallel *Consumer Expert Delphi* study. Phase 4 involved finalization of consensus statements.

**Results:**

Topic experts from Australia (*n* = 62, 92.5%) and New Zealand (*n* = 5, 7.5%) with a mean ± SD age of 45.7 ± 11.8 years participated in Phase 2; 38 (56.7%) were women, 38 (56.7%) were health professionals and 27 (40.3%) were researchers/academics. In Phase 2, 15 of 18 (83.3%) statements on sarcopenia prevention, screening, assessment, management and future research were accepted with strong agreement. The strongest agreement related to encouraging a healthy lifestyle (100%) and offering tailored resistance training to people with sarcopenia (92.5%). Forty‐seven experts participated in Phase 3; 5/6 (83.3%) revised statements on prevention, assessment and management were accepted with strong agreement. A majority of experts (87.9%) preferred the revised European Working Group for Sarcopenia in Older Persons (EWGSOP2) definition. Seventeen statements with strong agreement (>80%) were confirmed by the Task Force in Phase 4.

**Conclusions:**

The ANZSSFR Task Force present 17 sarcopenia management and research recommendations for use by health professionals and researchers which includes the recommendation to adopt the EWGSOP2 sarcopenia definition in Australia and New Zealand. This rigorous Delphi process that combined evidence, consumer expert opinion and topic expert consensus can inform similar initiatives in countries/regions lacking consensus on sarcopenia.

## Introduction

Sarcopenia is a skeletal muscle condition characterized by low muscle mass, strength, and physical performance.^1^, ^2^ Sarcopenia prevalence increases with age, present in up to 29% of community‐dwelling older adults and higher in those who are hospitalized, have multi‐morbidity, frailty, or who are using residential aged care (nursing home) services.^3^, ^4^, ^5^, ^6^ Sarcopenia is associated with increased risk of falls and fractures,^7^ health care costs^8^ and mortality.^9^, ^10^ However, knowledge on sarcopenia among health professionals remains poor^11^ and few organizations have protocols to diagnose and treat sarcopenia.^11^ Public awareness is also disproportionately low compared with other age‐related health issues,^12^ despite willingness of older adults to engage in sarcopenia treatment.^13^

In 2016, sarcopenia was assigned an International Classification of Disease code (ICD‐10‐CM M62.84) which was recognized in Australia in 2019.^14^, ^15^ Despite advances in knowledge about sarcopenia, there is currently no global consensus definition and little evidence of knowledge translation into clinical practice. Numerous operational definitions for sarcopenia have been developed since the term's inception in 1989.^16^ These definitions have been either consensus‐based^1^, ^17^, ^18^, ^19^, ^20^, ^21^, ^22^, ^23^ or data‐driven (i.e., the application of machine learning to produce optimal variables and cut‐points).^24^, ^25^ Recent definitions are detailed in Data S1. Agreement between these definitions is poor,^26^ resulting in variable prevalence estimates and conflicting treatment decisions.^27^

In 2017, the Australian and New Zealand Society for Sarcopenia and Frailty Research (ANZSSFR) formed a Task Force on Diagnostic Criteria for Sarcopenia (now the ‘Sarcopenia Diagnosis and Management Task Force’, henceforth referred to as ‘Task Force’). The objective of the Task Force was, through a modified Delphi method, to reach consensus on the preferred operational definition of sarcopenia in Australia and New Zealand (ANZ) for clinical and research applications.^28^ The Delphi method is a consensus‐building, iterative process that explores agreement and disagreement among participants to achieve representative consensus among potentially discordant groups.^29^ The Task Force reached consensus to adopt and promote the original European Working Group on Sarcopenia in Older People (EWGSOP1) definition,^20^ but the currency of that recommendation was short‐lived; in 2018, the EWGSOP presented a revised consensus definition of sarcopenia (EWGSOP2),^1^ and this was followed by an update to the Asian Working Group for Sarcopenia (AWGS) consensus on sarcopenia diagnosis and treatment. In late 2020, the Sarcopenia Diagnostic and Outcomes Consortium (SDOC) presented a new data‐driven definition of sarcopenia^25^ and in 2022 the South Asia Working Action Group on SARCOpenia (SWAG‐SARCO) also published a consensus document.^23^

This paper describes a Task Force initiative to establish consensus on sarcopenia together with clinical and research guidelines for use in ANZ, the target population for which was adults aged ≥55 years and/or with medical co‐morbidities. Two parallel Delphi processes were undertaken with the aim to develop statements with accompanying levels of evidence to guide ANZ‐based health professionals and researchers on sarcopenia screening, diagnosis, assessment, prevention, management, and future research. The opinions of (1) Task Force members plus other researchers and health professionals (‘topic experts’) and (2) experts with lived experience of sarcopenia or related healthcare experiences (‘consumer experts’) were determined.^30^

## Methods

This study comprised three components: (i) development of statements and questions by two Task Force members (J. Z. and D. S.) who systematically reviewed the literature and presented evidence to the Task Force to test face validity of statements and questions; (ii) a three‐Phase modified Delphi study involving consumer experts (‘Consumer Expert Delphi’); and (iii) a four‐phase modified Delphi study involving topic experts (‘Topic Expert Delphi’) from ANZ.

**Figure 1:**
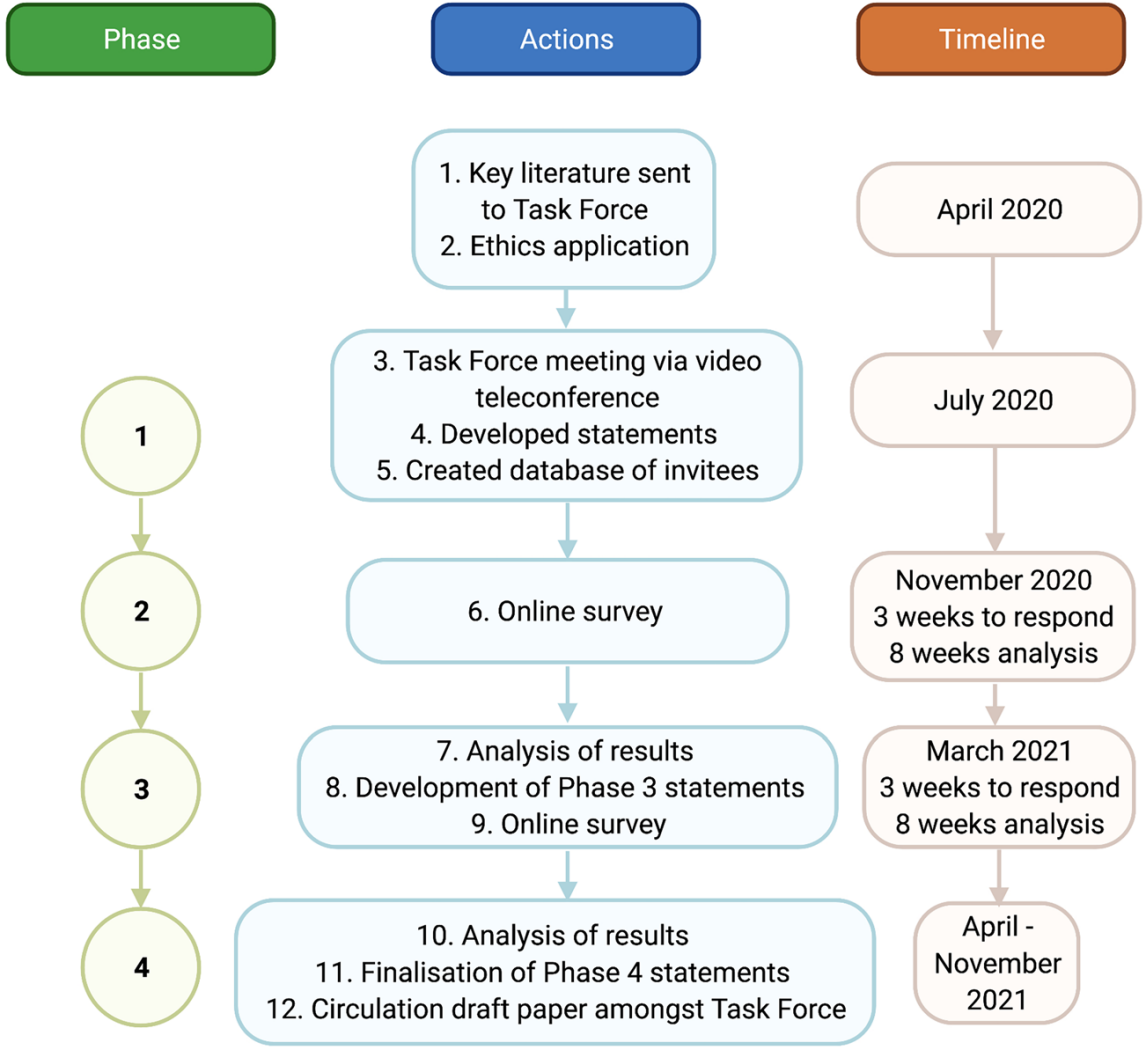
Flow chart of Topic Expert Delphi. Preceding Phase 1, a systematic literature search was undertaken to develop a Supplement of key literature. Phase 1 was a videoconference meeting of the Task Force, including presentations from Task Force members on sarcopenia definition progress and history of the Delphi method. Statements were debated and re‐drafted, and a decision to incorporate consumer feedback through a parallel Consumer Expert Delphi was made. Phase 2 involved a presentation at an ANZSSFR educational event (D. S.), including invitation and promotion of the Delphi study. Those who expressed interest and consented to the study were invited by email link to participate in the Topic Expert Delphi survey. The Phase 3 online survey was developed in response to the results and analysis from both Topic Expert and Consumer Expert Delphi studies in Phase 2. Phase 4 involved analysis of Phase 3 results and the finalization of statements among task force members.

In Phase 1, an online vote was undertaken, and consensus achieved to conduct the parallel Consumer Expert Delphi, and the format of the Topic Expert Delphi (Phases 2 to 4) was defined as described in Figure 1. Phase 2 findings from the Consumer Expert Delphi informed Phase 3 of the Topic Expert Delphi^30^; detailed outcomes of this process are published separately.^30^ This study was approved by Monash Health Human Research Ethics Committee (ERM 64175) and complied with ethical standards.^31^ All participants provided written informed consent electronically.

### Task Force, Panel and Topic Expert participants

Members of the original Task Force (*n* = 24),^28^ comprising clinicians, healthcare providers and researchers, were invited to participate via email. Recognizing the need to increase diversity and geographical representation, five topic experts were nominated to join the Task Force by existing members. Task Force members (*n* = 29) contributed to Phases 1 to 4 of the Topic Expert Delphi, completing topic expert surveys in Phases 2 (*n* = 67) and 3 (*n* = 47). JZ and DS developed surveys with feedback from Task Force members who tested face validity prior to circulation.

To obtain a wide range of perspectives, email invitations were sent to ANZ clinical and academic groups with potential interest in sarcopenia for distribution to members. Eleven national organizations were contacted (Data S2). Advertisements were also posted on Task Force members' and societies' social media accounts (e.g., Twitter and LinkedIn) and the ANZSSFR website. Invitations and advertisements directed potential participants to the online informed consent form. Links to Phase 2 and Phase 3 online surveys (Qualtrics) were forwarded to email addresses of individuals who provided informed consent to participate in the study prior to Phase 2.

### The modified Delphi method

Recommended procedures for a Delphi study were adhered to^29^ with the methodology and scope of the Task Force's previously published Delphi study was expanded upon.^28^ The Delphi method is iterative; thus, Phases 2 to 4 of the study were informed by findings from the prior Phases. Raw and synthesized results from Phases 2 and 3 were provided to participants consistent with best practice.^29^ This process of providing transparent, anonymous feedback between Delphi rounds is intended to increase the likelihood of achieving a consensus in the subsequent round by reducing the range of responses and the number of outliers. Participants were provided with preambles for each question and statement, providing context and references to relevant literature (Data S3 and S4).

#### Searching the evidence and statement development

Authors J. Z. and D. S. undertook a systematic search of the literature on 24 June 2020. The search strategy comprised: (i) publication libraries of JZ and DS and (ii) PubMed database searches with search term combinations ‘sarcopenia’ AND ‘prevention’ OR’ ‘screening’ OR ‘diagnosis’ OR ‘management’ OR ‘treatment’ (Data S5). Key references were provided to Task Force members in Phase 1 (Data S6).

Evidence informing development and finalization of statements was scrutinized applying both National Health and Medical Research Council (NHMRC) and GRADE Evidence to Decision (EtD) frameworks.^32^, ^33^, ^34^ Questions were developed using the ‘PICO’ (Population, Intervention, Comparison, Outcome) format. The evidence base was examined to determine strength and certainty of the evidence answering the PICO question and informing the statement. ‘Strength’ of evidence was determined by factors including benefits and harms, feasibility, acceptability, accuracy, and evidence quality.^33^ ‘Certainty’ of evidence was assessed by considering imprecision, risk of bias, inconsistency, publication bias and indirectness.^33^ Some statements were modified based on the parallel Consumer Expert Delphi results^30^ in accordance with NHMRC standards regarding consumer input.^32^ Statements were then classified as one or more of (i) evidence‐based recommendation (EBR); (ii) consensus‐based recommendation (CBR); and (iii) practice‐point (PP) based on criteria (Data S7) arising from NHMRC and GRADE EtD frameworks.^32^, ^33^, ^34^, ^35^ Task Force members confirmed these classifications in Phase 4 with reference to literature (Data S6).

#### Statistical analyses

Analyses were performed using a pre‐specified strategy.^28^, ^36^ Multiple choice questions were analysed descriptively and text responses by inspection (there were insufficient text responses for thematic analysis). An 11‐point Likert scale ranging from *strongly disagree* (0) to *strongly agree* (10) accompanied each statement. Statement was classified as having: strong agreement (>80% respondents scoring ≥7 or ≤3), moderate agreement (70% to 80% respondents scoring ≥7 or ≤3) or low agreement (<70% respondents scoring ≥7 or ≤3). Statements with strong agreement were accepted. Statements with moderate agreement were analysed to determine whether moderate agreement was due to heterogeneity (median comparison, Wilcoxon scores rank test) or dispersion (interquartile range ≥4). Statements with low or no agreement were rejected and excluded from subsequent phases.

## Results

This four‐phase modified Topic Expert Delphi process produced 17 recommendations by consensus (strong agreement >80%). Accepted and rejected statements are presented below and results for all questions are presented in Data S8.

### Phase 1: Video‐teleconference of task force

On 18 July 2020, 29 Task Force Members attended a three‐hour videoconference. Draft Phase 2 questions and statements were debated and re‐drafted until consensus (>80%) was achieved on Phase 2 online survey content. Consensus (>80%) was also achieved (via online vote) to incorporate a parallel Consumer Expert Delphi^30^ to inform the *Topic Expert Delphi*.

### Phase 2: Online survey

#### Phase 2 participant characteristics

Seventy‐six experts consented to participate in the Topic Expert Delphi, and of these, 67 (88%) experts (mean age ± SD, 45.7 ± 11.8 years) completed the Phase 2 survey (Table 1), including 38 (56.7%) women and 38 (56.7%) clinicians. Twenty‐seven participants (40.3%) stated their primary roles as researchers/academics. All states and territories in Australia (*n* = 62, 92.5%) were represented except Tasmania and Northern Territory, and 5 (7.5%) experts were from New Zealand.

**Table 1:** Topic expert characteristics in Phases 2 and 3

Characteristic	Sub‐category	Phase 2	Phase 3
		*n* = 67	*n* = 47
Mean age, years (SD)		45.7 (11.8)	44.9 (13.8)
Gender, *n* (women, %)		38 (56.7)	25 (53.2)
Median survey completion time, minutes, (IQR)		28.4 (15.4, 46.4)	14.4 (8.9, 23.9)
Location, *n* (%)	New Zealand	5 (7.5)	3 (6.4)
	Victoria	30 (44.8)	24 (51.6)
	New South Wales	16 (23.9)	11 (23.4)
	South Australia	6 (9.0)	3 (6.4)
	Queensland	5 (7.5)	2 (4.3)
	Western Australia	4 (6.0)	3 (6.4)
	Australian Capital Territory	1 (1.5)	1 (2.1)
	Tasmania	0 (0)	0 (0)
	Northern Territory	0 (0)	0 (0)
Background and descent, *n* (%)	Caucasian/European	53 (79.1)	39 (83.0)
	Asian	7 (10.4)	4 (8.5)
	Aboriginal Australian	1 (1.5)	1 (2.1)
	Middle Eastern	1 (1.5)	0 (0)
	Prefer not to say	5 (7.5)	3 (6.4)
Primary role, *n* (%)	Clinician (health professional)	38 (56.7)	21 (44.7)
	Dietitian	9 (13.4)	7 (14.9)
	Physiotherapist	6 (9.0)	2 (4.3)
	Geriatrician	3 (4.5)	2 (4.3)
	Nurse	2 (3.0)	1 (2.1)
	Other^a^	9 (13.4)	6 (12.8)
	Not specified	9 (13.4)	3 (6.4)
	Academic	22 (32.8)	21 (44.7)
	Professor	9 (13.4)	9 (19.1)
	Associate Professor	5 (7.5)	3 (6.4)
	Research Fellow (e.g., post‐doctoral and senior)	4 (6.0)	4 (8.5)
	Lecturer	2 (3.0)	3 (6.4)
	Not specified	2 (3.0)	2 (3.0)
	Researcher	5 (7.5)	4 (4.3)
	Manager, Aged Care Facility	2 (3.0)	1 (2.1)

IQR = interquartile range (25%–75%). SD = standard deviation.

a Clinician subclass: chiropractor, endocrinologist, exercise physiologist, geriatrician/rheumatologist, hepatologist/gastroenterologist, occupational therapist, physician, psychiatrist, rehabilitation.

### Phase 2 statements

**Figure 2:**
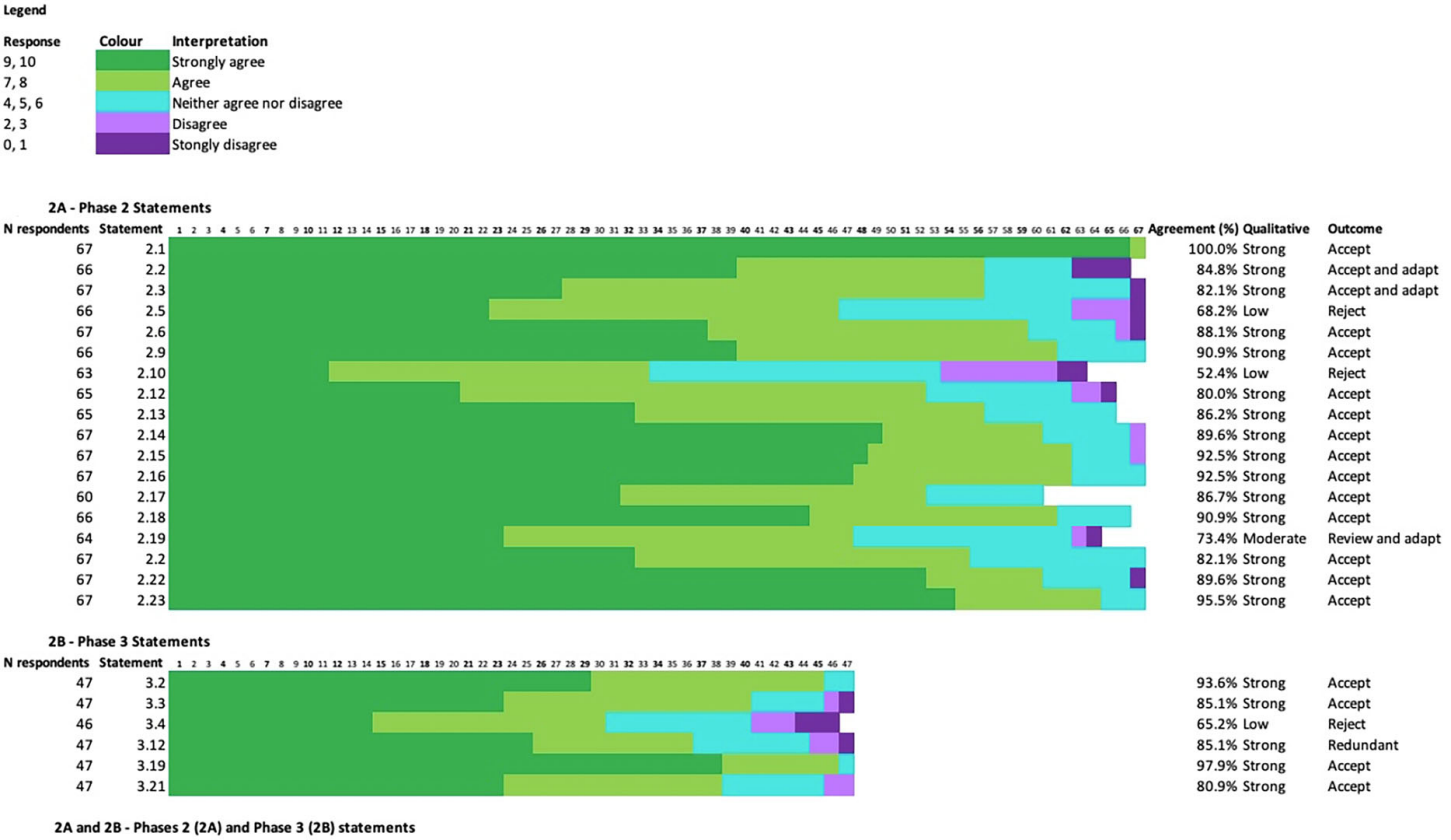
Phases 2 (A) and Phase 3 (B) statements. Graphical representation of level of agreement with each statement in Phases 2 and 3. Strong agreement: >80% respondents answering ≥7 or ≤3; moderate agreement: 70% to 80%; low agreement: <70%. ‘Legend; response’ describes the number out of 10 the respondent selected and the colour this response is represented by in each row. Note that all sequential numbers (**2.1** to **2.23** and **3.2** to **3.21**) are not present due to questions being imbedded in the survey represented by numbers not contained in Figure 2A,B. Non‐responses to particular questions were not included in calculation of agreement. ‘Redundant’ refers to a statement that while accepted, was included in the case that a definition of sarcopenia did not reach agreement—see 3.12 below. ‘*’ denotes rejected statements. Statements: **2.1**: A healthy lifestyle, including balanced diet, adequate protein intake, and regular exercise should be encouraged in adults of all ages; **2.2**: Person‐centred physical and dietary interventions, developed with an accredited healthcare professional (or degreed, NZ), are recommended for those with health conditions or states, such as frailty, likely to increase the risk of sarcopenia in adults; **2.3**: Adults aged 65 years and older, Aboriginal, Torres Strait Islander, Pacific Islander and Maori Elders aged 55 years and older, or those with conditions or circumstances that may increase the risk of sarcopenia at a younger age, should be screened for sarcopenia annually or after the occurrence of a major health event; 2.5*: Application of diagnostic criteria for sarcopenia should be used instead of any screening tool, where the required equipment and expertise for diagnosis is available, in those meeting the criteria in statement **3**; **2.6**: Adults screened as positive for possible sarcopenia should be assessed by an accredited health professional (or degreed, NZ) for further assessment to confirm sarcopenia; **2.9**: Low muscle mass is an important feature of sarcopenia; 2.10*: DXA should be used to determine low lean mass when diagnosing sarcopenia; **2.12**: In the absence of equipment required for sarcopenia diagnosis, or when physical limitations (e.g., hand arthritis) preclude some active testing, the presence of muscle weakness or slowness (low usual gait speed) makes sarcopenia probable; **2.13**: Cultural, ethnic and physical ability differences for normal and low muscle strength, physical performance and body composition measures should be considered in the application of diagnostic cut‐points for sarcopenia; **2.14**: Accredited healthcare professionals (or degreed, NZ) should provide an accessible explanation of sarcopenia, including provision of informative material, to those diagnosed with sarcopenia to support engagement in self‐determined health behaviours; **2.15**: All persons with sarcopenia should be offered resistance‐based training by an accredited healthcare professional (or degreed, NZ), tailored to the individuals' abilities and preferences; **2.16**: All adults with sarcopenia should be screened/assessed for malnutrition using validated tools; **2.17**: Total protein intake of 1–1.5 g/kg/day should be considered for older adults with sarcopenia, excepting those with significant kidney disease defined by an eGFR of <30 mL/min/1.73 m^2^; **2.18**: Clinicians should consider referring persons with sarcopenia to a dietitian for the development of a dietary and protein optimization plan; **2.19**: Optimization of dietary and protein intake may only be beneficial for persons with sarcopenia when combined with a physical activity intervention, such as resistance exercise; **2.20**: Persons with sarcopenia should be assessed at least annually following diagnosis, with additional assessment following any major health event; **2.22**: The standardization of a sarcopenia definition and cut‐points for diagnosis and management is recommended across Australia and New Zealand; **2.23**: Local and international collaborations, laboratory‐based studies, registries, randomized controlled trials and translational studies are recommended to improve management of and outcomes for people living with sarcopenia and translation of evidence into clinical practice; **3.2**: Person‐centred physical and dietary interventions, developed with an accredited healthcare professional (or degreed, NZ), are recommended for adults with health conditions known as likely to increase the risk of sarcopenia, such as frailty; **3.3**: Provided that adequate resources and training are available and assessment is acceptable to the individual, adults at risk of sarcopenia should be assessed for sarcopenia annually or after the occurrence of a the risk of major health event sarcopenia in adults; 3.4*: SARC‐F, with or without calf circumference measurement, is the preferred screening tool for sarcopenia in Australia and New Zealand; **3.12**: The ANZSSFR supports the use of either the revised EWGSOP2 definition, the SDOC definition, or if appropriate based on patient characteristics, the revised AWGS definition. Clinicians and researchers should clearly document the definition applied and aim for consistent application across their organization(s); **3.19**: Optimization of energy and protein intake is likely to be beneficial for all persons with sarcopenia, but benefits may be greatest when combined with a physical activity intervention, such as resistance exercise; **3.21**: The ANZSSFR recommends clinicians undertake a consultation of 30–60 min duration with persons with or at risk of sarcopenia, which would include assessments described by the BASIC (Basic Assessment Sarcopenia Items for Completion).

Experts responded to **18** statements (Figure 2A) in Phase 2. Fifteen statements were accepted with strong agreement (>80%). One statement had moderate agreement (70%–80%) and was revised for reconsideration in Phase 3. Two statements were rejected with low agreement (<70%), specifically; “Dual Energy Xray Absorptiometry (DXA) should be used to determine low lean mass when diagnosing sarcopenia” (52.4% agreement); and “application of diagnostic criteria for sarcopenia should be used instead of any screening tool, where the required equipment and expertise for diagnosis is available” (68.2% agreement). Free text responses were reviewed by the panel, contributing to minor amendments to wording of two already‐accepted statements, which were re‐presented in Phase 3 (Figure 2A; Statements **2.2** and **2.3**).

#### Phase 2 questions

**Figure 3:**
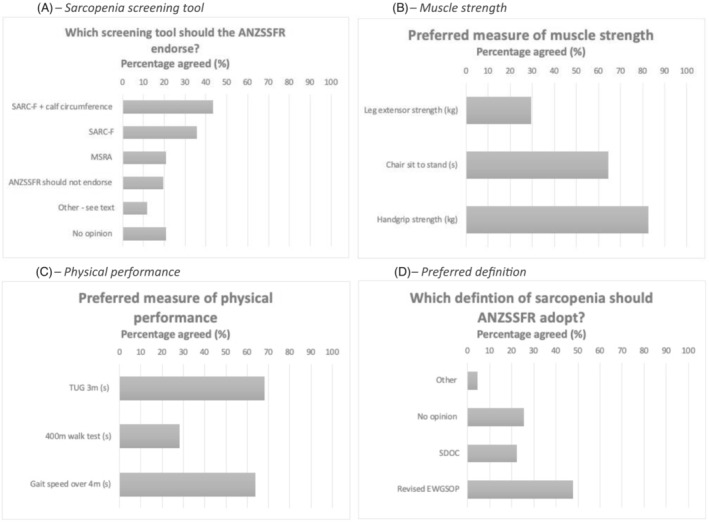
(A–D) Phase 2 question results on screening, muscle strength, physical performance, and sarcopenia definition. *N* = 67 topic experts. (A–C) Topic experts could select more than one response. (D) Topic experts could select only one response. ANZSSFR = Australian and New Zealand Society for Sarcopenia and Frailty Research. SDOC = Sarcopenia Diagnostic and Outcomes Consortium. EWGSOP2 = Revised European Working Group for Sarcopenia in Older Persons. MSRA = Mini Sarcopenia Risk Assessment. TUG = Timed‐Up‐And‐Go test over 3 m.

Topic experts were able to select one or more responses for five questions on sarcopenia screening and assessment (Figure 3A–D and Table 2). No clearly preferred screening tool for sarcopenia was identified (Figure 3A), with ‘SARC‐F’ (*n* = 24, 35.8%) and ‘SARC‐F with calf circumference’ (*n* = 29, 43.3%) most commonly reported. Regarding muscle strength assessment, handgrip strength was the preferred measure (*n* = 52, 82.0%), followed by chair‐sit‐to‐stand (*n* = 40, 64.5%), and leg extensor strength (*n* = 16, 29.6%) (Figure 3B). There was no distinctly preferred measure of physical performance, with the Timed‐Up‐And‐Go (TUG) test over 3 m the most preferred (*n* = 41, 68.3%), followed by normal gait speed over 4 m (*n* = 39, 63.9%), and 400 metre walk test (*n* = 17, 28.3%) (Figure 3C). There was no majority preferred definition of sarcopenia, with the EWGSOP2 definition most commonly preferred (*n* = 32, 47.8%), followed by ‘no opinion’ (*n* = 17, 25.4%) and the SDOC definition (*n* = 15, 22.4%) (Figure 3D).

**Table 2:** Phase 2 topic expert opinion on important elements in an assessment of people with sarcopenia

Rank	Assessment	Count	Percentage^a^
1	Falls history	62	93.9%
2	Sarcopenia diagnostic measures (depending on definition, e.g., grip strength and walking speed)	60	90.9%
2	Functional status (ability to undertake ADLs/iADLS)	60	90.9%
4	Nutritional assessment	58	87.9%
5	Fracture history	52	78.8%
6	Physical activity levels (e.g., S‐IPAQ)	48	72.7%
7	Overall quality of life	47	71.2%
8	Current medications	45	68.2%
9	Multimorbidity and comorbidity	44	66.7%
10	Cognition	43	65.2%
11	Self‐rated health	42	63.6%
12	Social support	41	62.1%
13	Pain	36	54.5%
14	Adherence with treatment recommendations	34	51.5%
15	Mood assessment	31	47.0%
16	Depression (e.g., GDS)	29	43.9%
17	Anxiety (e.g., GAI)	20	30.3%

ADL = Activities of Daily Living. iADLs = Instrumental Activities of Daily Living. GDS = Geriatric Depression Scale. GAI = Geriatric Anxiety Index. S‐IPAQ = Short International Physical Activity Questionnaire.

^a^ 66 out of 67 survey respondents completed this question.

Topic experts were requested to identify essential elements of assessment for older persons who may have sarcopenia (Table 2). Falls history was most selected (*n* = 62, 93.9%), followed equally by sarcopenia diagnostic criteria and functional status (*n* = 60, 90.9%). Least commonly selected were assessments of adherence with treatment recommendations (*n* = 34, 51.5%), mood (*n* = 31, 47.0%), depression (*n* = 29, 43.9%) and anxiety (*n* = 20, 30.3%).

### Consumer Expert Delphi results and impact on Phase 3 statements

Consumer expert opinion differed from topic expert opinion on two statements related to sarcopenia diagnosis and assessment.^30^ Consumer experts favoured at least annual assessments, had no preference on which type of health professional diagnosed sarcopenia, and preferred a consultation duration of 30–60 min.^30^ Consumer experts viewed mood problems as very important outcomes of sarcopenia, whereas topic experts did not. In addition, consumer experts identified resistance exercise as their preferred activity to prevent and treat sarcopenia and were willing to undertake dietary modifications.^30^ These findings were incorporated into statements **2**, **3**, **10** and **14** in the Phase 3 Topic Expert Delphi survey.

### Phase 3: Online survey

#### Phase 3 participant characteristics

Forty‐seven experts completed the survey in Phase 3 (Table 1), of whom 25 (53.2%) were women with a mean age ± SD, 44.9 ± 13.8 years, with equal representation from clinicians (*n* = 21, 44.7%) and academics (*n* = 21, 44.7%). All Australian states and territories (*n* = 44, 93.6%) were represented except for Tasmania and the Northern Territory, and three (6.4%) topic experts were from New Zealand.

#### Phase 3 statements

Topic experts responded to six statements in Phase 3 (Figure 2B). Five statements (**2**, **3**, **5**, **10** and **14**) were accepted with strong agreement (>80%). One statement was rejected with low agreement (65.2%): ‘SARC‐F, with or without calf circumference measurement, is the preferred screening tool for sarcopenia in Australia and New Zealand’.

#### Phase 3 questions

**Figure 4:**
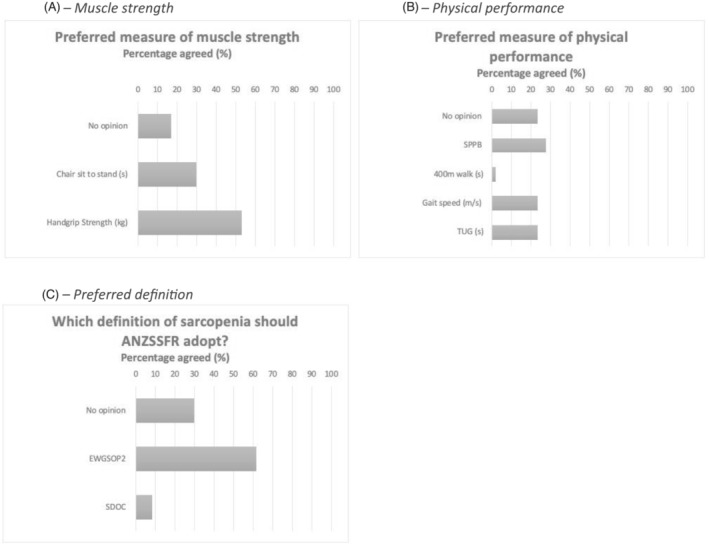
(A–C) Phase 3 question results on screening, muscle strength, physical performance, and sarcopenia definition. *n* = 47; topic experts could select only one response. ANZSSFR = Australian and New Zealand Society for Sarcopenia and Frailty Research. EWGSOP2 = Revised European Working Group for Sarcopenia in Older Persons. SDOC = Sarcopenia Diagnostic and Outcomes Consortium. SPPB = Short Physical Performance Battery. TUG = Timed‐Up‐And‐Go test over 3 m.

Topic experts responded to three questions (Figure 4A–C) in Phase 3, where only one response option for each question was allowed. The preferred measure of muscle strength was handgrip strength (*n* = 25, 53.2%), followed by chair‐sit‐to‐stand (*n* = 14, 29.8%), and ‘no opinion’ (*n* = 8, 17.0%) (Figure 4A). Excluding ‘no opinion’ (*n* = 8) from the analysis showed handgrip strength (64.1%) was strongly preferred compared with chair‐sit‐to‐stand (35.9%). There was no clearly preferred measure of physical performance, with SPPB (*n* = 13, 27.7%) slightly preferred, over TUG test over 3 m (*n* = 11, 23.4%), normal gait speed over 4 m (*n* = 11, 23.4%), and ‘no opinion’ (*n* = 11, 23.4%) (Figure 4B).

The majority of topic experts preferred the EWGSOP2 definition of sarcopenia (*n* = 29, 61.7%), followed by ‘no opinion’ (*n* = 14, 29.8%) and then the SDOC definition (*n* = 4, 8.5%) (Figure 4C). Excluding ‘no opinion’ (*n* = 14) from the analysis increased the percentage of those in favour of EWGSOP2 (87.9%) compared with SDOC (12.1%).

### Phase 4: Confirmation of recommendations

Twenty‐nine Task Force members reviewed findings from Phases 2 and 3 of the Delphi study and confirmed the classification of statements. Of **17** statements, 2 were classified as evidence‐based recommendations, 8 as consensus‐based recommendations, and 7 as practice points (Table 3).

**Table 3:** Australian and New Zealand Society for Sarcopenia and Frailty Research (ANZSSFR) Sarcopenia Diagnosis and Management Task Force final statements and classification of recommendation

Number	Statement	Agreement (%)	EBR	CBR	PP
*Prevention*					
1	A healthy lifestyle, including balanced diet, adequate protein intake, and regular exercise should be encouraged in adults of all ages.	100			
2	Person‐centred physical and dietary interventions, developed with an accredited healthcare professional (or degreed, NZ), are recommended for adults with health conditions known as likely to increase the risk of sarcopenia, such as frailty.	93.6			
*Screening*					
3	Provided that adequate resources and training are available, and assessment is acceptable to the individual, adults at risk of sarcopenia should be assessed for sarcopenia annually or after the occurrence of a major health event.	85.1			
4	Adults screened as positive for possible sarcopenia should be assessed by an accredited health professional (or degreed, NZ) for further assessment to confirm sarcopenia.	88.1			
*Diagnosis and assessment*					
5	The ANZSSFR endorses the use of the revised European Working Group for Sarcopenia in Older People (EWGSOP2) in clinical and research settings,^a^ including its validation in Australia and New Zealand.	85.1			
6	Low muscle mass is an important feature of sarcopenia.	90.9			
7	In the absence of equipment required for sarcopenia diagnosis, or when physical limitations (e.g., hand arthritis) preclude some active testing, the presence of muscle weakness or slowness (low usual gait speed) makes sarcopenia probable.	80.0			
8	Cultural, ethnic and physical ability differences for normal and low muscle strength, physical performance and body composition measures should be considered in the application of diagnostic cut‐points for sarcopenia.	86.2			
9	Persons with sarcopenia should be assessed at least annually following diagnosis, with additional assessment following any major health event.	82.1			
10	The ANZSSFR recommends clinicians undertake a consultation of 30–60 min duration with persons with or at risk of sarcopenia, which could include assessments described by the BASIC (Basic Assessment Sarcopenia Items for Completion).^b^	80.9			
11	The standardization of a sarcopenia definition and cut‐points for diagnosis and management is recommended across Australia and New Zealand.	89.6			
*Management*					
12	Accredited healthcare professionals (or degreed, NZ) should provide an accessible explanation of sarcopenia, including provision of informative material, to those diagnosed with sarcopenia to support engagement in self‐determined health behaviours.	89.6			
13	All persons with sarcopenia should be offered resistance‐based training by an accredited healthcare professional (or degreed, NZ), tailored to the individuals' abilities and preferences.	92.5			
14	Optimization of energy and protein intake is likely to be beneficial for all persons with sarcopenia, but benefits may be greatest when combined with a physical activity intervention, such as resistance exercise.	97.9			
15	Clinicians should consider referring persons with sarcopenia to a dietitian for the development of a dietary and protein optimization plan.	90.9			
16	Total protein intake of 1–1.5 g/kg/day should be considered for older adults with sarcopenia, excepting those with significant kidney disease defined by an eGFR of <30 mL/min/1.73 m^2^.	86.7			
*Research*					
17	Local and international collaborations, laboratory‐based studies, registries, randomised controlled trials and translational studies are recommended to improve management of and outcomes for people living with sarcopenia and translation of evidence into clinical practice.	95.5			

CBR = Consensus‐based recommendation. EBR = Evidence‐based recommendation. PP = Practice point.

^a^ Caveats to this recommendation are insufficient agreement to endorse a screening tool or imaging technique, addressed in *Discussion*.

^b^ The Basic Assessment Sarcopenia Items for Completion include (i) sarcopenia diagnostic measures; (ii) comorbidity assessment; (iii) medication history; (iv) falls and fracture history; (v) functional status; (vi) nutritional assessment; (vii) physical activity levels; (viii) social support assessment; (ix) quality of life and self‐rated health; and (x) cognition and mood assessment. Refer to Data S8 for more details.

## Discussion

This modified Delphi process conformed to pre‐specified standards for guideline development,^29^, ^32^ and consulted a range of topic and consumer experts across ANZ. We achieved strong agreement on **17** statements, forming the basis of recommendations for sarcopenia prevention, screening, diagnosis and assessment, management, and research in ANZ. These recommendations do not replace clinical judgement but can assist clinical decision‐making in line with evolving best practice and consumer values and preferences. This Delphi process could inform similar initiatives in other regions lacking consensus on sarcopenia.

Recent definitions based on opinion^1^, ^22^, ^23^ or analyses of large datasets^25^ have failed to establish international consensus on the diagnostic criteria for sarcopenia, and guidelines informed by health professionals are lacking. Further, consumers (or those with lived experience of sarcopenia), the most crucial focus of sarcopenia care and research, have hitherto not contributed to guidelines and consensus statements. This prompted the ANZSSFR Task Force to expand upon our original Delphi study completed in 2018.^28^

Here, we discuss elements underpinning the **17** statements. We aim to provide clarity for clinicians and researchers on the application of statements in clinical and research settings to community dwelling adults aged ≥55 years and/or with medical co‐morbidities.

### Prevention

Statement **1** promotes encouraging healthy diet and physical activity across the lifespan. This statement reflects epidemiological associations between healthy behaviours and improved health across the lifespan.^37^ Statement **1** was the only statement which achieved 100% agreement among respondents, suggesting topic experts uniformly view prevention and health promotion as important aspects of care. Reducing sedentary behaviour,^37^ and achieving a balanced diet^38^ are reflected in this statement. Statement **2** recognizes that those living with comorbidities, such as frailty, have higher risk of having or developing sarcopenia^4^ and that physical and dietary interventions aligned with individuals' values and preferences should be considered by interdisciplinary health professionals to reduce this risk.^39^ In the Consumer Expert Delphi, respondents highlighted a range of activities that would be acceptable to them to prevent sarcopenia, from resistance exercise to tai chi, and dietary modification.^30^

### Screening, diagnosis and assessment

Statements **9** and **10** are practice points guiding clinicians to consider the frequency, duration, and content of sarcopenia assessments. Statement **9** recommends assessments at least annually, or when a major health event (e.g., fall, fracture, and hospital admission) occurs. The recommendation of an annual assessment is consistent with the International Conference for Frailty and Sarcopenia Research (ICFSR) sarcopenia clinical practice guidelines^40^ and considers the progressive nature of sarcopenia coupled with acute declines in muscle mass, strength and physical performance resulting from acute illness [S1, S2]. The recommended consultation duration of 30–60 min is based upon findings of the Consumer Expert Delphi.^30^ Recent work has suggested methods for implementation of sarcopenia guidelines in hospital settings [S3]; however, further research is required to determine how assessments can be implemented within existing and future models of care in the community.

The Basic Assessment Sarcopenia Items for Completion (BASIC) (Data S9) is a compilation of 10 items preferred by consumer and topic experts from both Delphi studies.^30^ These items are not intended to replace clinical or comprehensive geriatric assessments but may guide clinicians when undertaking assessment of a person with, or at risk of sarcopenia.

Measures of muscle strength, physical performance and lean mass that define ‘normal’ from ‘sarcopenic’ vary across the lifespan, between sexes, races and between those of different socio‐economic status [S4]. In Phase 1 the Task Force acknowledged the challenges of non‐representative data and the lack of diversity often present in clinical studies for defining sarcopenia cut‐points [S5]. Generating a regional‐specific definition of sarcopenia is beyond the scope of this work and the Task Force recognized that with low sarcopenia knowledge and uptake among health professionals [S6], provision of yet another definition of sarcopenia may compound uncertainty, complicate research progress, and reduce knowledge translation into clinical practice.

In Phase 3, there was strong agreement on the adoption of the EWGSOP2 definition of sarcopenia in ANZ (Statement **5**). As illustrated in Data S1, EWGSOP2 uses the approach; find cases; assess; confirm; and (determine) severity (termed F‐A‐C‐S).^1^ EWGSOP2 diagnostic cut‐points are also listed in Data S1. Statement **8** supports, in principle, adjusted cut‐points for measures of strength, physical performance and surrogates of muscle mass where deemed clinically appropriate. For example, during the assessment of sarcopenia in a person of Asian descent, the health professional may consider the application of cut‐points from the updated AWGS consensus.^22^ Specific to our region, there is a paucity of data on normative values of strength, physical performance, and muscle mass for ANZ's first peoples (Aboriginal, Torres Strait Islander, and Māori) which may impact the application of existing cut‐points to these priority populations. The Task Force recognizes the need for further research in this area to better understand consumer perspectives, as broadly addressed in Statement **17**.

#### Case finding (screening)

To *find cases*, EWGSOP2 recommends the use of SARC‐F [S7] or clinical suspicion. In Phase 2, Statement **5**, “Application of diagnostic criteria for sarcopenia should be used instead of any screening tool, where the required equipment and expertise for diagnosis is available …”, was rejected with low agreement indicating health professionals support the use of screening tools for sarcopenia‐case finding despite recent literature suggesting that application of sarcopenia diagnostic criteria without screening is appropriate [S8]. However, in Phase 3, use of SARC‐F, with or without calf circumference, was rejected with low agreement and this may be related to the poor accuracy of SARC‐F and its modified versions in identifying those at risk of sarcopenia [S9]. As such the Task Force recommends that although screening tools such as SARC‐F may be used for sarcopenia screening consistent with the EWGSOP2 algorithm, it is also acceptable to use clinical suspicion (e.g., falls, feeling weak, weight loss, and reduced mobility [refer to Statement **2**, Table 3]), as a prompt for further assessment as highlighted in Statement **4**. It is also important for health professionals to consider the local availability of adequate training and resources in proceeding to assessment (Statement **3**), and potentially to facilitate patient access to centres with appropriate expertise and equipment where necessary [S10].

#### Diagnosis (probable and confirmed sarcopenia)

According to EWGSOP2, low muscle strength (determined by handgrip strength or chair‐sit‐to‐stand test) makes sarcopenia ‘probable’; this was supported by topic experts in Statement **7**. The Task Force endorses handgrip strength to measure muscle strength, noting experts preferred handgrip strength over chair‐sit‐to‐stand test.

The Delphi method facilitates anonymous, diverse opinions on specific questions that may not be easily answered by analytical techniques or in clinical trials. Both an attribute and challenge of this method is that aspects of a statement may be disputed, yet the overall statement accepted. This is reflected in the rejection of ‘DXA should be used to determine low lean mass when diagnosing sarcopenia’ due to low agreement, but strong agreement that ‘low muscle mass is an important feature of sarcopenia.’ Importantly, DXA estimates *lean mass*, which is not equivalent to, *muscle mass*. Experts appears to recognize increasing evidence that DXA lean mass may not be a reliable indicator of functional outcomes, supported by the SDOC findings that ‘lean mass measured by DXA should not be included in the definition of sarcopenia” [S11].

The EWGSOP2 definition of sarcopenia requires the presence of low muscle quality or quantity (determined by DXA, Bioelectrical Impedance Analysis (BIA), Computerized Tomography, or Magnetic Resonance Imaging).^1^ However, in Australia and New Zealand, public funding for and access to these techniques are minimal or absent, except in research settings. Further, the value of the more readily available techniques (DXA and BIA) to predict negative outcomes in those with sarcopenia as compared with more accurate but clinically unavailable measures of muscle quantity, such as the D_3_‐Creatine dilution technique, remains debated [S11–13].

In practice, where measures of muscle quality and quantity are not possible, and if sarcopenia is deemed *probable* (based on measurement of hand grip strength where possible), we endorse consideration of progressing to recommendations listed under ‘Management’ (Table 3). Topic experts agreed that either muscle weakness (e.g., low handgrip strength) or slowness (e.g., low gait speed over 4 m) makes sarcopenia *probable* (Statement **7**). This contrasts the EWGSOP2 definition, which defines *probable* sarcopenia as low muscle strength by either handgrip strength or chair‐sit‐to‐stand test^1^; however, gait speed is a recommended assessment of physical performance in both AWGS2 and SDOC definitions.^22^, ^25^

To determine sarcopenia *severity*, the EWGSOP2 recommends measurement of physical performance by one of: normal gait speed over 4 m; SPPB; TUG; or 400 metre walk test.^1^ In the Consumer Expert Delphi, a majority of respondents (*n* = 24, 88%) reported that they would be willing to undergo all assessments and tests deemed necessary to diagnose sarcopenia. Phase 3 results of the Topic Expert Delphi reflect the diversity of opinions on these measures with respondents identifying no clear preference for one measure over another (excepting 400 metre walk test which had low support). However, there are criticisms of using multiple methods to measure physical performance, including variable prevalence estimates and classification errors that can occur within and between individuals.^26^, ^27^

Statement **11** highlights the importance of standardization of measures of muscle strength and physical performance. Operationally, this means that within institutions and when following up individual patients, the same measures should be used over time to track progress and identify meaningful change. In the context of sarcopenia, clinically meaningful change is represented by a change in muscle strength or physical performance over time that may have clinical implications [S14]. While the Topic Expert Delphi did not address this, a change in normal gait speed of ≥0.1 m/s or an SPPB improvement of 0.5–1.0 points may represent clinically meaningful change [S14,15]. Health professionals should be aware that change in handgrip strength after an exercise intervention is not a reliable indicator of clinically meaningful change [S16] and so other measures (e.g., gait speed, SPPB) should be used for longitudinal monitoring.

**Figure 5:**
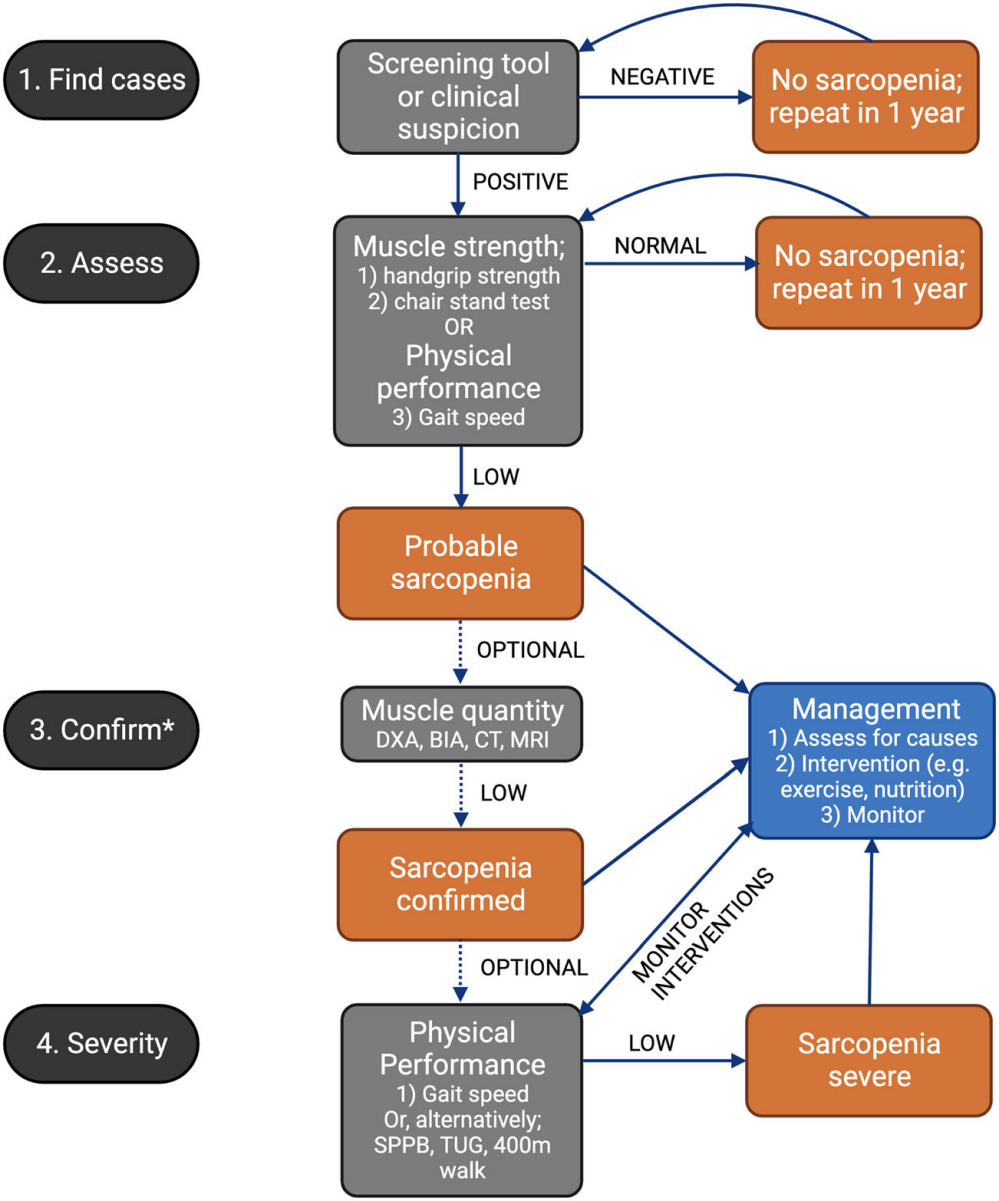
Modified EWGSOP2 diagnostic algorithm based on Delphi findings. *Step ‘3. Confirm’ is an optional step which may be limited by access to imaging resources. BIA = bioelectrical impedance analysis. CT = computed tomography. DXA = dual energy X‐ray absorptiometry. MRI = magnetic resonance imaging. SPPB = Short Physical Performance Battery. TUG = Timed‐Up‐And‐Go test over 3 m. The recommendations for management of sarcopenia do not vary across sarcopenia categories (e.g., probable/confirmed/severe).

Based on the findings of this Delphi process, the Task Force has modified the EWGSOP2 diagnostic algorithm for health professionals and researchers in ANZ (Figure 5). The algorithm is modified to provide recommendations for different settings according to availability of resources (Statement **3**). In step 1 (Find cases), potential sarcopenia cases are identified using an established screening tool or clinical suspicion. Step 2 (Assess) involves determining presence of probable sarcopenia via assessment of muscle strength, preferably using handgrip dynamometry as preferred by topic experts (Figure 4A), but alternatively via the sit‐to‐stand test if dynamometry is not available. In the absence of capacity to measure muscle strength via handgrip strength (i.e., in low‐resource settings), gait speed over 4 m may also be used in Step 2, given our experts recognized that presence of low usual gait speed makes sarcopenia probable (Statement **7**). In settings without resources for muscle quantity assessment, a finding of low muscle strength, or slowness, is sufficient to diagnose probable sarcopenia and commence management strategies. Indeed, it is important to note that recommendations for management of sarcopenia do not vary across categories of sarcopenia.

Proceeding to assessment for ‘confirmed sarcopenia’ (Step 3: Confirm) is recommended in settings with capacity to assess muscle quantity given confirmed sarcopenia is diagnosed when low muscle strength and low lean/muscle mass are present. However, given EWGSOP2 does not recommend gait speed to define probable sarcopenia, we do not recommend proceeding to Step 3 if this measurement was used in Step 2 (i.e., assessments would cease with a diagnosis of probable sarcopenia where gait speed was used in Step 2: Assess). Assessments for ‘severe sarcopenia’ can optionally be performed in those with confirmed sarcopenia; severe sarcopenia is diagnosed if poor physical performance (e.g., by gait speed <0.8 m/s) is identified in addition to low muscle strength and low muscle quantity (Step 4). It is therefore not possible to progress to assessment of sarcopenia severity without the prior step of confirming the sarcopenia diagnosis via muscle quantity; however, the physical performance assessments recommended in Step 4 are nonetheless of value for patients with probable and other categories of sarcopenia as they may be used to monitor and adapt interventions (as described earlier).

### Management

Critical to treatment success is consumer understanding, engagement and adherence coupled with knowledgeable health professionals to develop clear management programs with consumers [S17]. The importance of the consumer‐health professional relationship is highlighted in Statement **12** which was modified prior to Phase 3 to reflect the Consumer Expert Delphi findings.^30^

The most clear and unequivocal recommendations arising from our study (as supported by EBR classification) are recommendations regarding diet and exercise for those living with sarcopenia (Statements **13** and **14**). Consistent with the recent International Exercise Recommendations in Older Adults consensus guidelines, we recommend all persons with sarcopenia be offered resistance‐based exercise tailored to individual abilities and preferences [S18],^40^ particularly given there are presently no pharmaceutical treatments for sarcopenia. The prescription of exercise should be considered, discussed, and prescribed, with progress monitored like any other medical treatment [S18]. This may be best achieved by referral to an Accredited Exercise Physiologist or similarly qualified health professional. Access to these services is limited in certain settings (e.g., rural and remote locations), and therefore consideration of innovative modes of health care delivery (e.g., telehealth) could be made.^30^

Statement **14** recognizes that the effect of adequate protein and energy intake may be optimized by resistance exercise, particularly in those with low habitual protein (energy) intake [S19]. Given the specific skill set required to comprehensively assess and optimize dietary intake, and intervene where indicated, we recommend health professionals consider referring patients with sarcopenia to a dietitian (e.g., Accredited Practising Dietitian in Australia and New Zealand) (Statement **15**). The specific amount of protein recommended for older adults with sarcopenia remains contested, with conflicting evidence [S20]. As such, we recommend (Statement **16**) clinicians consider targeting a total protein intake of 1.0–1.5 g/kg/day in persons with sarcopenia, ideally ≥1.2 g/kg spread evenly throughout the day, with or without supplementation. The exception to this is in those with stage IV chronic kidney disease (estimated glomerular filtration rate <30 mL/min/1.73 m^2^) [S21], in whom clinical judgement should be applied.

### Research

Despite rapid growth in sarcopenia research globally, our study has highlighted many knowledge gaps in the field. Statement **17** highlights the importance of sarcopenia research across the translational sphere to advance the field and improve the lives of those living with sarcopenia. The Task Force envisage, with targeted research action and collaboration, many of the consensus‐based recommendations and practice points in our study could become evidence‐based recommendations.

### Strengths and limitations

Our study was strengthened by involvement of a wide range of experts across ANZ, coupled with input from the Consumer Expert Delphi.^30^ The Topic Expert Delphi adhered to reporting recommendations for Delphi studies^29^ and presented clear definitions of levels of evidence,^32^, ^33^ which has been noted as lacking in guidelines across ANZ [S22]. Selection bias was mitigated by non‐exclusive inclusion criteria for topic experts, and response bias was addressed *a priori* by high agreement thresholds, anonymity and each response being considered equal. To maximize the accessibility of the survey for health professionals without expertise on operational definitions and diagnosis of sarcopenia, and to minimize the impact of satisficing (i.e., where respondents answer not in reflection of their beliefs or knowledge but to complete a survey in a timely manner), we presented a ‘no opinion’ option for questions on the preferred sarcopenia definition and measures of muscle strength and physical performance. Our study was limited by moderate participant numbers, although participant numbers were twice that of the previous Delphi study^28^ and likely reflect low levels of awareness and expertise in sarcopenia among health professionals. Attrition was noted between Phase 2 (*n* = 67) and Phase 3 (*n* = 47), which may have affected the findings. Finally, there was limited representation from New Zealand and some Australian states and territories, which may affect generalizability of the findings to those regions.

## Conclusion

This modified Delphi method informed by evidence, and topic and consumer expert opinions, generated 17 consensus recommendations on sarcopenia prevention, screening, diagnosis and assessment, management, and future research. This process may inform similar processes in other regions/countries lacking consensus on sarcopenia. Adoption of these recommendations by policy makers, health professionals and scientists can increase translation of sarcopenia knowledge into clinical and research practice in Australia and New Zealand, with the potential to improve the lives of people living with sarcopenia. However, such adoption is only likely to occur through targeted initiatives which promote public and clinical knowledge of sarcopenia.

## Funding

DS is supported by an Australian National Health and Medical Research Council Investigator Grant (GNT1174886). GD is supported by grants from the Australian Medical Research Future Fund (APP2005987). JAP has recently received funding from the NHMRC (APP1162867), MRFF (APP1199726), Deakin University, Amgen, Department of Health and Human Services (DHHS), and the Norman Beischer Foundation. JL is supported by a National Heart Foundation Future Leader Fellowship (ID: 102817). JZ is supported by an Australian Government Research Training Program (TRP) Scholarship. MG is supported by an NHMRC project grant (APP1099173). MS is supported by a Royal Perth Hospital Career Advancement Fellowship (CAF 130/2020), an Emerging Leader Fellowship and project grant from the Western Australian Future Health and Innovation Fund. SI has received funding from Dairy Australia, California Dairy Research Foundation, National Dairy Council, Aarhus University Hospital and Danish Dairy Research Foundation, Fonterra Co‐operative Group Ltd, Dutch Dairy Association, Dairy Council of California, Dairy Farmers of Canada, the Centre national interprofessionnel de l'economie laitiere, University of Melbourne, Austin Hospital Medical Research Foundation and Sir Edward Dunlop Medical Research Foundation. SP is supported by an NHMRC Postgraduate Scholarship, grant number 2003179. RV is supported by the NHMRC CRE 1102208 and Hospital Research Foundation.

## Conflicts of interest

ABM has received speaker and consulting fees from Abbott, Nutricia, AstraZeneca, Novartis. GD is a member of the Scientific Advisory Board of TSI, Abbott and Amgen and has received speaker/consulting fees from Amgen, Abbott and TSI. MG has received research funding from Bayer Pharma, Novartis, Weight Watchers, Lilly, Otsuka and speaker's honoraria from Bayer Pharma, Besins Healthcare, and Amgen. RMD reports a grant form Fonterra Co‐operative Group Ltd, honoraria for presentations from Abbott Australia and Nutricia Research and to serve as a member of an expert advisory committee. RV has previously received education and honorarium from the following Abbott, Nestle and Nutricia. SI has received speaker/consulting fees from Abbott, UK Dairy Council, European Milk Forum, Nestle Health Science and the Israel Milk Board.

## Supporting information


**Table S1**. Recent operational definitions of sarcopenia

**Data S1.** Minimum list of invitees

**Data S2.** Phase 2 Online Survey

**Data S3.** Phase 3 Online Survey

**Table S2.** Population, Intervention, Comparison, Outcome (PICO) questions and GRADE assessments

**Data S4.** Key literature

**Table S3.** Classification of statements by agreement, and strength and certainty of evidence

**Figure S1.** Results Phases 2 and 3

**Data S5.** The Basic Assessment Sarcopenia Items for Completion (BASIC)

**Data S6.** References S1 to S22
