# Supplementary material for: Consensus guidelines for sarcopenia prevention, diagnosis and management in Australia and New Zealand
Source: J Cachexia Sarcopenia Muscle. 2022 Nov 9;14(1):142–56. doi: 10.1002/jcsm.13115 (PMC9891980; doi:10.1002/jcsm.13115)
Supplement: Supplementary file 1 — Table S1. Recent operational definitions of sarcopenia [file JCSM-14-142-s005.docx]

**Supplement 1 - Recent operational definitions of sarcopenia**

| **Component** | **Cut-points** |
| --- | --- |
| **Initial European Working Group on Sarcopenia in Older People [**20] **adopted by ANZSSFR in 2018 [**28]  Sarcopenia = Low lean mass and low muscle strength and low physical performance | |
| Low muscle (lean) quantity  Low muscle strength  Low physical performance | ALM using whole-body DXA:  A*djusted for height (m^2^)* Men: < 7.26kg/m^2^ Women: < 5.50kg/m^2^  Hand grip strength using dynamometer:  Men: < 30kg  Women: < 20kg  Men and women over 4m course:  Gait speed: ≤ 0.8 m/s |
| **Revised European Working Group on Sarcopenia in Older People [**1]  Probable sarcopenia = Low muscle strength  Sarcopenia = Low muscle strength AND low muscle quality or quantity  Severe sarcopenia = Low muscle strength AND low muscle quality or quantity AND low physical performance | |
| Low muscle quantity  Low muscle strength  Low physical performance | ASM adjusted for height (m^2^) using whole-body DXA: Men: <7.0 kg/m^2^ Women: <5.5 kg/m^2^ ASM using DXA: Men: <20.0 kg  Women: <15.0 kg  Hand grip strength using dynamometer:  Men: <27 kg  Women: <16 kg  Chair stand:  >15 s for 5 rises  Gait speed: ≤0.8m/s  SPPB: <8 point score  TUG: >12 s*  400 m walk test: non-completion of >6 min |
| **Asian Working Group for Sarcopenia: 2019 consensus update [**22]  Sarcopenia: Low ASM AND low muscle strength OR low physical performance Severe sarcopenia: Low ASM AND low muscle strength AND low physical performance | |
| Low muscle quantity  Low muscle strength  Low physical performance | ASM adjusted for height (m^2^) using whole-body DXA: Men: <7.0 kg/m^2^ Women: <5.54 g/m^2^ SMI adjusted for height (m^2^) using BIA: Men: <7.0 kg/m^2^  Women: <5.7 kg/m^2^  Hand grip strength using dynamometer:  Men: <28 kg  Women: < 18 kg  Gait speed: <1.0 m/s  Chair stand: >12 s for 5 rises  SPPB: <9 point score |
| **Sarcopenia Definition and Outcomes Consortium [**25]  Sarcopenia = Low muscle strength (grip strength) AND low physical performance (walking speed) | |
| Low muscle strength  Low physical performance | Hand grip strength using dynamometer:  Men: <35.5 kg  Women: <20 kg  Gait speed: <0.8 m/s |
| **South Asian Working Action Group on SARCOpenia (SWAG-SARCO)** [23]  Sarcopenia = Any two of sub-optimal muscle function, muscle strength and muscle mass | |
| Sub-optimal muscle function  Sub-optimal muscle strength  Sub-optimal muscle mass (or surrogates) | Gait speed: <0.8m/s (India); < 0.96 (Sri Lanka)  Chair stand: > 12 s for 5 rises  SPPB: < 9 point score  Men: < 27.5 kg  Women: < 18 kg  DXA ALM/height^2^:  Men: < 7.0 kg/m^2^  Women: < 5.7 kg/m^2^  BIA Fat free mass:  Men: < 7.0 kg/m^2^  Women: < 5.7 kg/m^2^ |

* The TUG cut-point identified by Cruz-Jentoft *et al* 2019 was >20s however the referenced article (Bischoff *et al* 2003) (63) recommend the cut-point >12s. ALM = Appendicular lean mass. ASM = Appendicular skeletal muscle mass. BIA = Bioelectrical impedance analysis. BMI = Body Mass Index. DXA = Dual energy x-ray absorptiometry. SMI = Skeletal Muscle Index. SPPB = Short physical performance battery. TUG = Timed Up and Go test.
