# Supplementary material for: Consensus guidelines for sarcopenia prevention, diagnosis and management in Australia and New Zealand
Source: J Cachexia Sarcopenia Muscle. 2022 Nov 9;14(1):142–56. doi: 10.1002/jcsm.13115 (PMC9891980; doi:10.1002/jcsm.13115)
Supplement: Supplementary file 2 — Data S1. Minimum list of invitees [file JCSM-14-142-s007.docx]

**Supplement 2 – Minimum list of invitees**

- Australasian Association of Gerontology (AAG)
- Australasian Society of Parenteral and Enteral Nutrition (AuSPEN)
- Australian and New Zealand Bone and Mineral Society (ANZBMS)
- Australian and New Zealand Falls Prevention Society
- Australian and New Zealand Society for Geriatric Medicine (ANZSGM)
- Australian and New Zealand Society for Sarcopenia and Frailty Research (ANZSSFR)
- Australian Health Policy Collaboration
- Australian Physiotherapy Association
- Australian Rheumatology Association (ARA)
- Clinical Oncology Society of Australia (COSA)
- Endocrine Society of Australia (ESA)
- Individuals suggested by Task Force Members
