# Supplementary material for: Consensus guidelines for sarcopenia prevention, diagnosis and management in Australia and New Zealand
Source: J Cachexia Sarcopenia Muscle. 2022 Nov 9;14(1):142–56. doi: 10.1002/jcsm.13115 (PMC9891980; doi:10.1002/jcsm.13115)
Supplement: Supplementary file 3 — Data S2. Phase 2 Online Survey [file JCSM-14-142-s006.docx]

**Supplement 3 – Phase 2 Online Survey**

**ANZZSFR Sarcopenia Diagnosis and Management Task Force**

**Phase 2 Expert Survey**

**Page 1**

**Plain Language Summary**

Sarcopenia is a condition which results in low muscle strength, size or physical performance (such as slow walking speed). Sarcopenia is common among older adults, particularly those with multiple medical problems and those living in residential care. Sarcopenia can contribute to a range of problems, including falls, fractures and death. Despite being an important condition, there is no agreed way to diagnose sarcopenia. There are no clear guidelines for health professionals to treat sarcopenia in Australia and New Zealand. The effects of sarcopenia that are actually important to people with the condition, such as falls or difficulty with household tasks, are unknown. It is also not known what sort of health assessment and treatment people with sarcopenia would be willing to undertake or what options are available to them. In this study, we will seek the opinions of a range of people including sarcopenia experts, health professionals, community members, people with sarcopenia and the caregivers of people living with sarcopenia to produce recommendations for researchers and clinicians in Australia and New Zealand working with people who live with or are at risk of sarcopenia.

**Page 2**

Thank you for your active participation in this important study on sarcopenia in Australia and New Zealand.

Please refer to our *Explanatory Statement* for more information. This is available on the ANZSSFR website at <https://anzssfr.org/> or can be sent to you via email. Email [dscott@monash.edu](mailto:dscott@monash.edu) to receive a copy of the explanatory statement.

Through this process of multi-stage process of consulting key stakeholders, such as yourself, we aim to establish consensus recommendations for the diagnosis and management of sarcopenia in Australia and New Zealand. Your participation in this survey, and a shorter Phase 3 survey, will greatly assist in achieving and promoting these consensus recommendations. We are most grateful for your valuable contribution.

The process of achieving consensus is through the “Delphi Method.” You have consented to involvement in Phases 2 and 3 of the Delphi method, which are short online surveys, where you are asked your opinion on a range of statements. Each survey will take less than 10 minutes. The statements presented are informed by the academic literature and confirmed in Phase 1. If you seek further reading regarding the statements, please email [dscott@monash.edu](mailto:dscott@monash.edu) to receive an Supplement of relevant readings. This is not compulsory to respond to the statements.

**The proposed timeline for the Delphi Method is:**

Phase 1

ANZSSFR Task Force Meeting – July 18^th^

Phase 2

Survey of key stakeholders across Australia and New Zealand

- Send out survey by mid-November
- Responses required 3 weeks after sending out survey

Phase 3

Review, collation, analysis and feedback of Phase 2 results

- Compile survey responses
- Analyse agreement and disagreement

Confirm and circulate Phase 3 survey of key stakeholders across Australia and New Zealand

- Send out survey by early February

Phase 4

Development of draft recommendations and academic manuscript and paper

- Analysis and presentation of results
- Circulation of finalised manuscript to all participants

**Page 3**

Thank you for agreeing to participate in our study. It is very important that you complete the questionnaires in each phase. The reliability of the results could be compromised if people drop out of the study before it is completed, because they feel that the rest of the group does not share their opinions. If people drop out because they feel their opinions are in the minority, the final results will overestimate how much the sample of participants agreed on this topic.

Your participation is voluntary and anonymous. Thank you for your valuable contribution.

**Completing the survey**

In addition to being asked demographic details, there are 23 statements. With each statement, you will be asked to rank the statement from 1 (strongly disagree) to 10 (strongly agree) on how much you agree with the statement. You can select, “I don’t have an opinion on this statement” if you are unsure of the content or how to answer. Free text will be available for you to comment with each question if you wish.

**Page 4**

*Participant demographic details*

Age

Gender M / F / Other (specify) / Prefer not to say

State/Country Drop-down list / prefer not to say
Do you identify as Y – Aboriginal, Torres Strait Islander, Maori / Pacific Islander /
 N - / Prefer not to say

Position / job title /role Drop-down list

**Page 5**

The following survey is mostly comprised of statements which will ask you to provide your level of agreement or disagreement with the statement. This is on a 10 point “Likert” scale ranging from strongly disagree (1) to strongly agree (10). “N/O” Stands for “No opinion on this statement.”

All statements except 4, 7, 8, 11 and 21 requests that you answer on the Likert scale. Items 4, 7, 8, 11 and 21 contain individual instructions on how to answer these questions.

Free text is available for each item.

| **Item** | **Preamble** | | | | | | | **Statement** | | | | |
| --- | --- | --- | --- | --- | --- | --- | --- | --- | --- | --- | --- | --- |
| 1 | The following statement refers to general lifestyle recommendations for all adults. Specific recommendations for those with or at risk of sarcopenia are explored in later statements. | | | | | | | **1) A healthy lifestyle, including balanced diet, adequate protein intake, and regular exercise should be encouraged in adults of all ages** | | | | |
|  | The following best represents my level of agreement with statement 1 | | | | | | | | | | | |
|  | Strongly disagree Strongly agree | | | | | | | | | | | |
|  | 1 | 2 | 3 | 4 | 5 | 6 | 7 | | 8 | 9 | 10 | N/O |
|  | Are there any other comments you wish to make? | | | | | | | | | | | |
| 2 | In the following statement, physical interventions describe exercise-based recommendations developed with the support of an accredited healthcare professional, tailored to the individual’s abilities and limitations. Dietary interventions describe modifications to dietary pattern and intake, which may or may not involve supplementation, with the support of accredited healthcare professionals, tailored to the individual’s values and preferences.  Health conditions or states that increase the risk of sarcopenia include, but are not limited to:  *Nutritional –* low protein intake; low energy intake; micronutrient deficiency; malabsorptive problems; anorexia (ageing, oral problems)  *Inactivity* – bed rest; immobility; deconditioning; low activity; sedentary lifestyle  *Disease –* bone and joint diseases (e.g. rheumatoid arthritis); cardiorespiratory disorders (e.g heart failure, COPD); metabolic disorders (e.g. diabetes); endocrine disorders (e.g. androgen deprivation); neurological disorders (e.g. stroke); cancer; liver and kidney disease  *Iatrogenic* – hospital admission; drug-related (e.g. corticosteroids).  “Accredited health professional” refers to a trained health practitioner registered under the Australian Health Practitioners Regulatory Authority (AHPRA) or under the jurisdiction of the New Zealand Health Practitioners Competence Assurance (HPCA) Act 2003 and falling within the respective and recognised scope of practice.” An additional provision is made for those with a degree in NZ, as exercise physiologists do no presently fall under the act. | | | | | | | **2) Person-centred physical and dietary interventions, developed with an accredited healthcare professional (or degreed, NZ), are recommended for those with health conditions or states, such as frailty, likely to increase the risk of sarcopenia in adults.** | | | | |
|  | The following best represents my level of agreement with statement 2 | | | | | | | | | | | |
|  | Strongly disagree Strongly agree | | | | | | | | | | | |
|  | 1 | 2 | 3 | 4 | 5 | 6 | 7 | | 8 | 9 | 10 | N/O |
|  | Are there any other comments you wish to make? | | | | | | | | | | | |
| 3 | The following statement refers to “older adults,” “screening,” “conditions or circumstances,” “major health events.”  In general, “older adults” are considered as 65 years or older. In certain population groups, owing to unique circumstances, older adults may be considered as 55 years and older. This includes Aboriginal, Torres Strait Islander, Pacific Islander and Maori Elders.  “Screening” in this instance refers to a general recommendation about assessments that may point towards looking further for sarcopenia. There are questions on screening specifics later in the survey.  “Conditions or circumstances” that may increase with risk of sarcopenia are described in the preamble for Statement 2.  “Major health events” describe an occurrence with one’s health that significantly (and possibly temporarily) disrupts how they carry out their usual day or duties. This may involve but is not limited to a fall, hospitalisation for any reason and illness limiting one to remain in bed for more than one day.  “Accredited health professional” refers to a trained health practitioner registered under the Australian Health Practitioners Regulatory Authority (AHPRA) or under the jurisdiction of the New Zealand Health Practitioners Competence Assurance (HPCA) Act 2003 and falling within the respective and recognised scope of practice.” An additional provision is made for those with a degree in NZ, as exercise physiologists do no presently fall under the act. | | | | | | | **3) Adults aged 65 years and older, Aboriginal, Torres Strait Islander, Pacific Islander and Maori Elders aged 55 years and older, or those with conditions or circumstances that may increase the risk of sarcopenia at a younger age, should be screened for sarcopenia annually or after the occurrence of a major health event** | | | | |
|  | The following best represents my level of agreement with statement 3 | | | | | | | | | | | |
|  | Strongly disagree Strongly agree | | | | | | | | | | | |
|  | 1 | 2 | 3 | 4 | 5 | 6 | 7 | | 8 | 9 | 10 | N/O |
|  | Are there any other comments you wish to make? | | | | | | | | | | | |
| 4 & 5 | “Screening” refers to the application of a test to a population which has no overt signs or symptoms of the disease in question, to detect disease at a stage when treatment is more effective. The screening test is used to identify people who require further investigation to determine the presence or absence of disease and is not primarily a diagnostic test (Reference - *Cancer Council Australia).*  With respect to sarcopenia, the term “case-finding” has been used instead of screening. This differs from screening in that case-finding may start when a person reports symptoms of sarcopenia, such as falls, feeling week, slowness, difficulty rising from a chair.  Here, we use the term “screening” to support the screening of persons who may not have symptoms of sarcopenia but who are at risk based on age or contributory health conditions.  Given that assessment of hand grip strength and normal walking speed may take less time in a consultation to complete than a questionnaire, it has been suggested by some that screening should be skipped and diagnosis sought instead. This notion informs statement 5.  *Resources / references will be supplied for the tools referenced.* | | | | | | | **4) The ANZSSFR should endorse which of the following screening tools for sarcopenia in those meeting the criteria in statement 3 (select all that apply)**   - **SARC-F** - **SARC-F and calf circumference** - **The Mini Sarcopenia Risk Assessment (MSRA)** - **ANZSSFR should not endorse a sarcopenia screening tool** - **Other, please specify**   **5) Application of diagnostic criteria for sarcopenia should be used instead of any screening tool, where the required equipment and expertise for diagnosis is available, in those meeting the criteria in statement 3.** | | | | |
|  | The following best represents my level of agreement with statement 5 | | | | | | | | | | | |
|  | Strongly disagree Strongly agree | | | | | | | | | | | |
|  | 1 | 2 | 3 | 4 | 5 | 6 | 7 | | 8 | 9 | 10 | N/O |
|  | Are there any other comments you wish to make? | | | | | | | | | | | |
| 6 | This question is dependent upon the selection results of statements 4 and 5. It assumes that there is a screening process.  If there is no screening tool recommended, or if screening is not recommended based on the phase 2 results, this statement will be removed in future phases.  “Accredited health professional” refers to a trained health practitioner registered under the Australian Health Practitioners Regulatory Authority (AHPRA) or under the jurisdiction of the New Zealand Health Practitioners Competence Assurance (HPCA) Act 2003 and falling within the respective and recognised scope of practice.” An additional provision is made for those with a degree in NZ, as exercise physiologists do no presently fall under the act. | | | | | | | **6) Adults screened as positive for possible sarcopenia should be assessed by an accredited health professional (or degreed, NZ) for further assessment to confirm sarcopenia** | | | | |
|  | The following best represents my level of agreement with statement 6 | | | | | | | | | | | |
|  | Strongly disagree Strongly agree | | | | | | | | | | | |
|  | 1 | 2 | 3 | 4 | 5 | 6 | 7 | | 8 | 9 | 10 | N/O |
|  | Are there any other comments you wish to make? | | | | | | | | | | | |
| 7 | There are different ways to measure muscle strength. These include hand grip strength, leg extensor strength and chair sit to stand. Some groups (EWGSOP2) describe chair sit to stand as measuring muscle strength. The statement below considers how easily a measure of muscle strength can be applied in a variety of clinical settings, including hospital, clinics and residential aged care facilities. This is not necessarily related to a definition of sarcopenia, but rather refers directly to how muscle strength is measured clinically.  In answering this question, “10” is your strongest recommendation, and “0” is your weakest recommendation. | | | | | | | **7) My recommendation for measuring muscle strength, which considers clinical application, timing, equipment, and utility in predicting negative outcomes, is (please score 1 to 5 – if you don’t wish to score an item, select N/A):**  **Handgrip strength 1 2 3 4 5 NA**  **5-time sit to stand test 1 2 3 4 5NA Leg extensor Strength 1 2 3 4 5 NA Other, please specify** | | | | |
| 8 | There are numerous measures available to assess physical performance. These are highlighted in the EWGSOP2 definition on measures to assess clinical performance.  In answering this statement, “1” is the strongest recommendation, and “5” is the weakest recommendation. | | | | | | | **8) My recommendation for measuring physical performance, which considers clinical application, timing, equipment, and utility in predicting negative outcomes, is (please score 1 to 5 – if you don’t wish to score an item, select N/A):**  **SPPB total score 1 2 3 4 5 NA Normal walking speed (4m) 1 2 3 4 5 NA**  **400m walking test 1 2 3 4 5 NA**  **Timed up and go test (3m) 1 2 3 4 5 NA**  **Other, please specify** | | | | |
| 9 | The original definition of sarcopenia in 1989 referred to “low lean mass” as being the hall mark of sarcopenia. Lean mass is an estimate of muscle mass measured by DXA, and includes connective tissues and is affected by body water. The most recent definition of sarcopenia by the SDOC does not include an estimate of muscle mass, but acknowledges that muscle is important – SDOC highlight that there might not be an accurate way of assessing muscle clinically at this stage. This statement seeks to clarify whether participants in the survey think muscle mass is important or not, in general. Lean mass has been included but this statement can be altered based on findings in other statements.  “Feature” in this statement refers to muscle mass having significantly involved in the development or symptoms of sarcopenia. | | | | | | | **9) Low muscle mass is an important feature of sarcopenia** | | | | |
|  | The following best represents my level of agreement with statement 9 | | | | | | | | | | | |
|  | Strongly disagree Strongly agree | | | | | | | | | | | |
|  | 1 | 2 | 3 | 4 | 5 | 6 | 7 | | 8 | 9 | 10 | N/O |
|  | Are there any other comments you wish to make? | | | | | | | | | | | |
| 10 | DXA is recommended in recent clinical practice guidelines (ICFSR) for the assessment of lean mass, which is a surrogate of muscle mass. In Australia and New Zealand, outside of research and hospital-based clinicals, measures of lean mass by DXA machines is not currently subsidised by the government. The recent EWGSOP2 definition includes DXA in its assessment, and SDOC does not. SDOC does not include DXA lean mass as it was not as strongly associated with walking speed as was handgrip strength, or negative outcomes.  This statement is general and does not refer to a specific definition of sarcopenia | | | | | | | **10) DXA should be used to determine low lean mass when diagnosing sarcopenia** | | | | |
|  | The following best represents my level of agreement with statement 10 | | | | | | | | | | | |
|  | Strongly disagree Strongly agree | | | | | | | | | | | |
|  | 1 | 2 | 3 | 4 | 5 | 6 | 7 | | 8 | 9 | 10 | N/O |
|  | Are there any other comments you wish to make? | | | | | | | | | | | |
| 11 | The most recent sarcopenia definitions are different in the diagnostic algorithm, cut-points and tests used to diagnosis sarcopenia, possible sarcopenia, probably sarcopenia and severe sarcopenia.  Background information and visual material will be provided to supplement the preamble, as per the draft Phase 2 survey. | | | | | | | **11) The ANZSSFR should adopt the following operational definition and associated cut-points of sarcopenia for use by clinicians and researchers in Australia and New Zealand:**   1. **Revised European Working Group for Sarcopenia in Older People (EWGSOP) definition** 2. **Sarcopenia Definition and Outcomes Consortium (SDOC) definition** | | | | |
|  | Are there any other comments you wish to make? | | | | | | | | | | | |
| 12 | There may appear to be overlap with one of more of the definitions of sarcopenia in with the following statement. The purpose of this statement is, for example, if agreement is established on a definition of sarcopenia that requires imaging or other tests not available to complete the diagnosis. In many rural, remote or resource-limited settings, further imaging or measurement tools such as hand grip dynamometers may not be available. This statement is intended to support accredited health professionals to consider recommendations to address sarcopenia even if individuals do not meet the full diagnostic criteria.  Other causes of low usual gait speed should also be considered if they co-occur, such as Parkinson Disease and other neurodegenerative disorders. | | | | | | | **12) In the absence of equipment required for sarcopenia diagnosis, or when physical limitations (e.g. hand arthritis) preclude some active testing, the presence of muscle weakness or slowness (low usual gait speed) makes sarcopenia *probable*** | | | | |
|  | The following best represents my level of agreement with statement 12 | | | | | | | | | | | |
|  | Strongly disagree Strongly agree | | | | | | | | | | | |
|  | 1 | 2 | 3 | 4 | 5 | 6 | 7 | | 8 | 9 | 10 | N/O |
|  | Are there any other comments you wish to make? | | | | | | | | | | | |
| 13 | The “normal” values for people of different ages, ethnicities, genders and physical abilities differ as shown across observational studies. Culture is a broad description, which can influence dietary, exercise and lifestyle behaviours and choices. | | | | | | | **13) Cultural, ethnic and physical ability differences for normal and low muscle strength, physical performance and body composition measures should be considered in the application of diagnostic cut-points for sarcopenia** | | | | |
|  | The following best represents my level of agreement with statement 13 | | | | | | | | | | | |
|  | Strongly disagree Strongly agree | | | | | | | | | | | |
|  | 1 | 2 | 3 | 4 | 5 | 6 | 7 | | 8 | 9 | 10 | N/O |
|  | Are there any other comments you wish to make? | | | | | | | | | | | |
| 14 | “Accredited health professional” refers to a trained health practitioner registered under the Australian Health Practitioners Regulatory Authority (AHPRA) or under the jurisdiction of the New Zealand Health Practitioners Competence Assurance (HPCA) Act 2003 and falling within the respective and recognised scope of practice.” An additional provision is made for those with a degree in NZ, as exercise physiologists do no presently fall under the act. | | | | | | | **14) Accredited healthcare professionals (or degreed, NZ) should provide an accessible explanation of sarcopenia, including provision of informative material, to those diagnosed with sarcopenia to support engagement in self-determined health behaviours** | | | | |
|  | The following best represents my level of agreement with statement 14 | | | | | | | | | | | |
|  | Strongly disagree Strongly agree | | | | | | | | | | | |
|  | 1 | 2 | 3 | 4 | 5 | 6 | 7 | | 8 | 9 | 10 | N/O |
|  | Are there any other comments you wish to make? | | | | | | | | | | | |
| 15 | There is strong evidence that resistance-based exercise (e.g. weight-lifting) can improve muscle strength and physical performance in people living with sarcopenia.  “Accredited health professional” refers to a trained health practitioner registered under the Australian Health Practitioners Regulatory Authority (AHPRA) or under the jurisdiction of the New Zealand Health Practitioners Competence Assurance (HPCA) Act 2003 and falling within the respective and recognised scope of practice.” An additional provision is made for those with a degree in NZ, as exercise physiologists do no presently fall under the act. | | | | | | | **15) All persons with sarcopenia should be offered resistance-based training by an accredited healthcare professional (or degreed, NZ), tailored to the individuals’ abilities and preferences** | | | | |
|  | The following best represents my level of agreement with statement 15 | | | | | | | | | | | |
|  | Strongly disagree Strongly agree | | | | | | | | | | | |
|  | 1 | 2 | 3 | 4 | 5 | 6 | 7 | | 8 | 9 | 10 | N/O |
|  | Are there any other comments you wish to make? | | | | | | | | | | | |
| 16 | Malnutrition has a strong association with sarcopenia. | | | | | | | **16) All adults with sarcopenia should be screened/assessed for malnutrition using validated tools.** | | | | |
|  | The following best represents my level of agreement with statement 16 | | | | | | | | | | | |
|  | Strongly disagree Strongly agree | | | | | | | | | | | |
|  | 1 | 2 | 3 | 4 | 5 | 6 | 7 | | 8 | 9 | 10 | N/O |
|  | Are there any other comments you wish to make? | | | | | | | | | | | |
| 17 | The evidence for protein supplementation in sarcopenia is “low” and current recommendations by the ICFSR are that there is a fine risk between benefits, risks and burdens. Given conflicting PROT-AGE and ICFSR guidelines, the recommendation has been expanded in order to reflect a "minimum" and a "safe maximum," rather than a narrow focus which may also be more difficult to apply in practice. Significant kidney disease is defined by the PROT-AGE study group as an estimated glomerular filtration rate (eGFR) of <30mL/min/1.73m^2^. | | | | | | | **17) Total protein intake of 1-1.5g/kg/day should be considered for older adults with sarcopenia, excepting those with significant kidney disease defined by an eGFR of <30mL/min/1.73m^2^.** | | | | |
|  | The following best represents my level of agreement with statement 17 | | | | | | | | | | | |
|  | Strongly disagree Strongly agree | | | | | | | | | | | |
|  | 1 | 2 | 3 | 4 | 5 | 6 | 7 | | 8 | 9 | 10 | N/O |
|  | Are there any other comments you wish to make? | | | | | | | | | | | |
| 18 | Accredited dietitians can develop person-centred dietary plans with persons living with sarcopenia. | | | | | | | **18) Clinicians should consider referring persons with sarcopenia to a dietitian for the development of a dietary and protein optimisation plan** | | | | |
|  | The following best represents my level of agreement with statement 18 | | | | | | | | | | | |
|  | Strongly disagree Strongly agree | | | | | | | | | | | |
|  | 1 | 2 | 3 | 4 | 5 | 6 | 7 | | 8 | 9 | 10 | N/O |
|  | Are there any other comments you wish to make? | | | | | | | | | | | |
| 19 | The evidence for combining adequate protein and energy intake with resistance exercise in persons with sarcopenia is low according to the ICFSR clinical practice guidelines. | | | | | | | **19) Optimisation of dietary and protein intake may only be beneficial for persons with sarcopenia when combined with a physical activity intervention, such as resistance exercise.** | | | | |
|  | The following best represents my level of agreement with statement 19 | | | | | | | | | | | |
|  | Strongly disagree Strongly agree | | | | | | | | | | | |
|  | 1 | 2 | 3 | 4 | 5 | 6 | 7 | | 8 | 9 | 10 | N/O |
|  | Are there any other comments you wish to make? | | | | | | | | | | | |
| 20 | This statement explores frequency of assessments for people with sarcopenia. At present, this statement suggests yearly however a concurrent consumer survey may influence the timeframe as consumers are being asked about their preferred timeframe of assessment.  “Major health events” describe an occurrence with one’s health that significantly (and possibly temporarily) disrupts how they carry out their usual day or duties. This may involve but is not limited to a fall, hospitalisation for any reason and illness limiting one to remain in bed for more than one day. | | | | | | | **20) Persons with sarcopenia should be assessed at least annually following diagnosis, with additional assessment following any major health event** | | | | |
|  | The following best represents my level of agreement with statement 13 | | | | | | | | | | | |
|  | Strongly disagree Strongly agree | | | | | | | | | | | |
|  | 1 | 2 | 3 | 4 | 5 | 6 | 7 | | 8 | 9 | 10 | N/O |
|  | Are there any other comments you wish to make? | | | | | | | | | | | |
| 21 | There currently is no recommended minimum set of requirements when undertaking an assessment of a person with sarcopenia. | | | | | | | **21) Please select as many of the following as you deem appropriate in a consultation where a person is being assessed for sarcopenia, and feel free to add additional assessments in the free text below**   - **Self-rated health** - **Overall quality of life** - **Falls history** - **Fracture history** - **Functional status (ability to undertake ADLs/iADLs)** - **Adherence with treatment recommendations** - **Sarcopenia diagnostic measures (depending on definition, e.g. grip strength, walking speed)** - **Pain** - **Nutritional assessment** - **Mood assessment** - **Physical activity levels (e.g. S-IPAQ)** - **Depression (e.g. Geriatric Depression Scale)** - **Anxiety (e.g. Geriatric Anxiety Index)** - **Multimorbidity and comorbidity** - **Current medications** - **Cognition** - **Social support** - **Other, please specify** | | | | |
| 22 | There is currently no consensus on guidelines for sarcopenia diagnosis and management in Australia and New Zealand. | | | | | | | **22) The standardisation of a sarcopenia definition and cut-points for diagnosis and management is recommended across Australia and New Zealand** | | | | |
|  | The following best represents my level of agreement with statement 22 | | | | | | | | | | | |
|  | Strongly disagree Strongly agree | | | | | | | | | | | |
|  | 1 | 2 | 3 | 4 | 5 | 6 | 7 | | 8 | 9 | 10 | N/O |
|  | Are there any other comments you wish to make? | | | | | | | | | | | |
| 23 | Research on sarcopenia has used different definitions making combining results challenging. Much research has also not been adopted into clinical practice. | | | | | | | **23) Local and international collaborations, laboratory-based studies, registries, randomised controlled trials and translational studies are recommended to improve management of and outcomes for people living with sarcopenia and translation of evidence into clinical practice** | | | | |
|  | The following best represents my level of agreement with statement 13 | | | | | | | | | | | |
|  | Strongly disagree Strongly agree | | | | | | | | | | | |
|  | 1 | 2 | 3 | 4 | 5 | 6 | 7 | | 8 | 9 | 10 | N/O |
|  | Are there any other comments you wish to make? | | | | | | | | | | | |

Are there any other comments you wish to make?

Thank you for completing this survey.

We will be in touch in the coming weeks with results and instructions regarding the next Phase of the Delphi study.
