# Supplementary material for: Consensus guidelines for sarcopenia prevention, diagnosis and management in Australia and New Zealand
Source: J Cachexia Sarcopenia Muscle. 2022 Nov 9;14(1):142–56. doi: 10.1002/jcsm.13115 (PMC9891980; doi:10.1002/jcsm.13115)
Supplement: Supplementary file 4 — Data S3. Phase 3 Online Survey [file JCSM-14-142-s004.docx]

**Supplement 4 – Phase 3 Online Survey**

**ANZSSFR Sarcopenia Diagnosis and Management Task Force**

**Phase 3 Expert Survey**

**Page 1**

You have been approached as you had previously provided your email and consented to participate in this important study on sarcopenia in Australia and New Zealand. Thank you to those who completed Phase 2 of this Delphi study. This shorter Phase 3 survey (5 to 10 minutes) more closely examines the areas of controversy or disagreement identified during Phase 2, and draws on the feedback from experts and consumers that was provided at that point to inform these current questions and statements.

**Plain Language Summary**

Sarcopenia is a condition which results in low muscle strength, size or physical performance (such as slow walking speed). Sarcopenia is common among older adults, particularly those with multiple medical conditions and those living in residential care. Sarcopenia can contribute to a range of conditions, including falls, fractures and death. Despite being an important condition, there is no agreed way to diagnose sarcopenia. There are no clear guidelines for health professionals to treat sarcopenia in Australia and New Zealand. The effects of sarcopenia that are actually important to people with the condition, such as falls or difficulty with household tasks, are unknown. It is also not known what sort of health assessment and treatment people with sarcopenia would be willing to undertake or what options are available to them. In this study, we will seek the opinions of a range of people including sarcopenia experts, health professionals, community members, people with sarcopenia and the caregivers of people living with sarcopenia to produce recommendations for researchers and clinicians in Australia and New Zealand working with people who live with or are at risk of sarcopenia.

**Page 2**

Please refer to our *Explanatory Statement* for more information. This is available on the Australian and New Zealand Society for Sarcopenia and Frailty Research (ANZSSFR) website at <https://anzssfr.org/> or can be sent to you via email. Email [dscott@monash.edu](mailto:dscott@monash.edu) to receive a copy of the explanatory statement.

Through this multi-stage process of consulting key stakeholders, such as yourself, we aim to establish consensus recommendations for the diagnosis and management of sarcopenia in Australia and New Zealand. Your participation will greatly assist in achieving and promoting these consensus recommendations. We are most grateful for your valuable contribution.

It is not compulsory to respond to the statements or questions. Your participation is voluntary and anonymous. Thank you for your valuable contribution.

**The timeline for the Delphi Method is:**

Phase 1


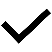
 ANZSSFR Task Force Meeting – July 18^th^ 2020

Phase 2


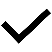
 Survey of key stakeholders across Australia and New Zealand


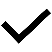
 Released survey mid-November 2020


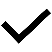
 Responses required by mid-December 2020

Phase 3


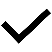
 Review, collation, analysis and feedback of Phase 2 results – January 2021


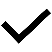
 Compile survey responses


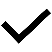
 Analyse agreement and disagreement


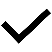
 Confirm and circulate Phase 3 survey of key stakeholders across Australia and New Zealand

- Send out survey by late February/early March 2021

Phase 4

Development of draft recommendations and academic manuscript and paper

- Analysis and presentation of results
- Circulation of finalised manuscript to all participants

**Page 3**

**Completing the survey**

In addition to being asked demographic details, there are 5 statements and 3 questions. With each statement, you will be asked to rank the statement from 0 (strongly disagree) to 10 (strongly agree) on how much you agree with the statement. You can select, “I don’t have an opinion on this statement” if you are unsure of the content or how to rank the statement. For each question, you will be provided with a selection of responses from which to choose and have the option of providing a comment if you wish.

**Page 4**

*Participant details
These questions are asked to help determine whether differences in responses might be related to participant characteristics (e.g. academics compared to clinicians).*

Age

Gender M / F / Other (specify) / Prefer not to say

State/Country Drop-down list / prefer not to say
Do you identify as Y – Aboriginal, Torres Strait Islander, Maori / Pacific Islander /
 N - / Prefer not to say

Position / job title /role Drop-down list

**Page 5**

**Statement 2 preamble**

**Original statement***Person-centred physical and dietary interventions, developed with an accredited healthcare professional (or degreed, NZ), are recommended for those with health conditions or states, such as frailty, likely to increase the risk of sarcopenia in adults.*

This statement received strong agreement (84.8%) in Phase 2, however compelling free text responses were considered to improve the readability and clarity of the statement. Please read the text below which explains the terminology in the newly presented statement.

In the following statement, “physical interventions” describe exercise-based recommendations (including those targeting balance) developed with the support of an accredited healthcare professional, tailored to the individual’s abilities and limitations. “Dietary interventions” describe modifications to dietary pattern and intake, which may or may not involve supplementation, with the support of accredited healthcare professionals, tailored to the individual’s values and preferences.

“Health conditions” that increase the risk of sarcopenia include, but are not limited to:

*Nutritional –* low protein intake; low energy intake; micronutrient deficiency; malabsorptive problems; anorexia (ageing, oral problems)

*Inactivity* – bed rest; immobility; deconditioning; low activity; sedentary lifestyle

*Disease –* bone and joint diseases (e.g. rheumatoid arthritis); cardiorespiratory disorders (e.g heart failure, COPD); metabolic disorders (e.g. diabetes); endocrine disorders (e.g. androgen deprivation); neurological disorders (e.g. stroke); cancer; liver and kidney disease

*Iatrogenic* – hospital admission; drug-related (e.g. corticosteroids).

“Accredited health professional” refers to a trained health practitioner registered under the Australian Health Practitioners Regulatory Authority (AHPRA) or under the jurisdiction of the New Zealand Health Practitioners Competence Assurance (HPCA) Act 2003 and falling within the respective and recognised scope of practice. An additional provision is made for those with a relevant degree in NZ, such as an exercise physiology degree, which do not presently fall under the act.

**Revised statement 2**

**Person-centred physical and dietary interventions, developed with an accredited healthcare professional (or degreed, NZ), are recommended for adults with health conditions known as likely to increase the risk of sarcopenia, such as frailty.**

**The following best represents my agreement with statement 2:**

| Strongly disagree | | | | Neither agree nor disagree | | | Strongly agree | | | |
| --- | --- | --- | --- | --- | --- | --- | --- | --- | --- | --- |
| 0 | 1 | 2 | 3 | 4 | 5 | 6 | 7 | 8 | 9 | 10 |
| I do not have an opinion on this statement | | | | | | | | | | |

Are there are any other comments you wish to make? _____________________________________

**Statement 3 preamble**

**Original statement**

*Adults aged 65 years and older, Aboriginal, Torres Strait Islander, Pacific Islander and Maori Elders aged 55 years and older, or those with conditions or circumstances that may increase the risk of sarcopenia at a younger age, should be screened for sarcopenia annually or after the occurrence of a major health event.*

This statement received strong agreement (82.1%) in Phase 2, however compelling free text responses were considered to improve the readability and clarity of the statement.

The following statement refers to “older adults,” “assessment,” and “major health events.”

In general, “older adults” are considered as 65 years or older. In some population groups, owing to different circumstances, “older adults” may be considered as 55 years and older. This may include Aboriginal, Torres Strait Islander, Pacific Islander and Maori Elders or those with conditions or circumstances that may increase the risk of sarcopenia.

“Conditions or circumstances” that may increase with risk of sarcopenia are described in the preamble for Statement 2.

“Health conditions” that increase the risk of sarcopenia include, but are not limited to:

*Nutritional –* low protein intake; low energy intake; micronutrient deficiency; malabsorptive problems; anorexia (ageing, oral problems)

*Inactivity* – bed rest; immobility; deconditioning; low activity; sedentary lifestyle

*Disease –* bone and joint diseases (e.g. rheumatoid arthritis); cardiorespiratory disorders (e.g heart failure, COPD); metabolic disorders (e.g. diabetes); endocrine disorders (e.g. androgen deprivation); neurological disorders (e.g. stroke); cancer; liver and kidney disease

*Iatrogenic* – hospital admission; drug-related (e.g. corticosteroids).

“Assessment” in this instance refers to a general recommendation about assessments that may point towards looking further for sarcopenia.

“Major health events” describe an occurrence with one’s health that significantly (and possibly temporarily) disrupts how they carry out their usual day or duties. This may involve but is not limited to a fall, hospitalisation for any reason and illness limiting one to remain in bed for more than one day.

“Accredited health professional” refers to a trained health practitioner registered under the Australian Health Practitioners Regulatory Authority (AHPRA) or under the jurisdiction of the New Zealand Health Practitioners Competence Assurance (HPCA) Act 2003 and falling within the respective and recognised scope of practice.” An additional provision is made for those with a relevant degree in NZ, such as an exercise physiology degree, which do not presently fall under the act.

**Revised statement 3**

**Provided that adequate resources and training are available and assessment is acceptable to the individual, adults at risk of sarcopenia should be assessed for sarcopenia annually or after the occurrence of a major health event**

**The following best represents my agreement with statement 3:**

| Strongly disagree | | | | Neither agree nor disagree | | | Strongly agree | | | |
| --- | --- | --- | --- | --- | --- | --- | --- | --- | --- | --- |
| 0 | 1 | 2 | 3 | 4 | 5 | 6 | 7 | 8 | 9 | 10 |
| I do not have an opinion on this statement | | | | | | | | | | |

Are there are any other comments you wish to make? _____________________________________

**Preamble statement 4**

There is no original “Statement 4,” rather this has been newly developed in Phase 3 based on responses to questions and statements in Phase 2.

In Phase 2, Statement 3, experts supported the concept that screening should be performed for sarcopenia (82.1% agreement). Statement 5, which suggested that it is appropriate to apply sarcopenia diagnostic measures initially (i.e. without the need for screening) received low agreement (68.2% agreement) and was therefore rejected. The strong agreement for Statement 6 (88.1% agreement) indicates that experts support the approach of screening for sarcopenia, followed by diagnosis to confirm sarcopenia in patients who screen positive. In Phase 2 Question 4, experts chose SARC-F and SARC-F with calf circumference as the two most preferred screening tools (35.8% and 43.3%, respectively).

*Resources / references will be supplied for the tools referenced.*

**Statement 4**

**SARC-F, with or without calf circumference measurement, is the preferred screening tool for sarcopenia in Australia and New Zealand.**

**The following best represents my agreement with statement 4:**

| Strongly disagree | | | | Neither agree nor disagree | | | Strongly agree | | | |
| --- | --- | --- | --- | --- | --- | --- | --- | --- | --- | --- |
| 0 | 1 | 2 | 3 | 4 | 5 | 6 | 7 | 8 | 9 | 10 |
| I do not have an opinion on this statement | | | | | | | | | | |

Are there are any other comments you wish to make? _____________________________________

**Preamble for question and statement 7**

This question relates to experts’ opinion on the preferred measure of muscle strength. More than one choice was offered in Phase 2. In Phase 2, experts chose handgrip strength (82.5%), followed by chair sit to stand (64.5%), and leg extensor strength (29.6%). It is noted that the current EWGSOP2 operational definition of sarcopenia recommends the use of either handgrip strength or chair sit to stand tests to assess muscle strength, but that prevalence of sarcopenia may vary substantially according to these different measurements. Accordingly, this question has been re-presented as a statement offering experts the opportunity to choose their preferred measure of either handgrip strength or chair sit to stand. If strong support is demonstrated for one measure, the ANZSSFR will recommend this measure in an effort to establish consistency in sarcopenia diagnosis approaches in Australia and New Zealand.

*Resources / references will be supplied for the tools referenced.*

<https://www.jamda.com/article/S1525-8610(18)30675-3/fulltext>

**Question and statement 7**

**Providing that access or uptake is not limited by patient-related factors or availability, the measure of muscle strength I recommend is;**

- **Handgrip strength by handheld dynamometer**
- **Chair sit to stand test**
- **I don’t have an opinion on this statement**

**Preamble for question and statement 8**

This question relates to experts’ opinion on the preferred measure of physical performance. More than one choice was offered in Phase 2. In Phase 2, experts chose timed up and go test (68.3%), followed by gait speed over 4m (63.9%), and 400m walk test (28.3%). The Short Physical Performance Battery (SPPB) was not included, but numerous experts requested its inclusion. As per the previous statement, the EWGSOP2 recommends either gait speed, SPPB, timed up and go test or 400m walk test as assessments for physical performance. If one assessment receives strong support from experts, then the ANZSSFR will recommend this measure in an effort to establish consistency in sarcopenia diagnostic approaches in Australia and New Zealand. This question has therefore been re-presented as a statement offering experts the opportunity to choose their preferred measure of these four measures of physical performance.

*Resources / references will be supplied for the tools referenced.*

**Question and statement 8**

**Providing that access or uptake is not limited by patient-related factors or availability, the measure of physical performance I recommend is;**

- **Timed Up and Go Test (TUGT)**
- **Gait speed over 4m**
- **400m walk test**
- **Short Physical Performance Battery (SPPB)**
- **I don’t have an opinion on this statement**

Are there are any other comments you wish to make? _____________________________________

**Preamble for question and statement 11**

In Phase 2, experts provided their opinion on which definition of sarcopenia the ANZSSFR should adopt. Experts chose EWGSOP2 (47.8%), followed by no preference/opinion (25.4%), followed by SDOC (22.4%).

The ANZSSFR Task Force acknowledges the diversity of opinions on this subject, and thus re-presents this question given there was no clear majority. Further, based on free text responses, the option for application of demographically appropriate sarcopenia definitions, such as the revised Asian Working Group definition, is included. Finally, should no clear majority be reached a statement is presented offering experts the opportunity to provide their opinion on how the ANZSSFR should progress with this issue.

*Resources / references will be supplied for the tools referenced.*

**Question 11**

**Except where patient characteristics suggest the use of the revised Asian Working Group definition of sarcopenia is more appropriate, the definition of sarcopenia I recommend is;**

- **Revised European Working Group for Sarcopenia in Older People (EWGSOP) definition**
- **Sarcopenia Definition and Outcomes Consortium (SDOC) definition**
- **I don’t have an opinion on this statement**

Should no clear majority be established in Question 11, the results based on responses to the statement below will apply.

**The ANZSSFR supports the use of either the revised European Working Group for Sarcopenia in Older People (EWGSOP2) definition, the Sarcopenia Definition and Outcomes Consortium (SDOC) definition, or if appropriate based on patient characteristics, the revised Asian Working Group definition for sarcopenia. Clinicians and researchers should clearly document the definition applied and aim for consistent application across their organisation(s).**

**The following best represents my agreement with this statement:**

| Strongly disagree | | | | Neither agree nor disagree | | | Strongly agree | | | |
| --- | --- | --- | --- | --- | --- | --- | --- | --- | --- | --- |
| 0 | 1 | 2 | 3 | 4 | 5 | 6 | 7 | 8 | 9 | 10 |
| I do not have an opinion on this statement | | | | | | | | | | |

Are there are any other comments you wish to make? _____________________________________

**Preamble for Statement 19**

**Original statement**

*Optimisation of dietary and protein intake may only be beneficial for persons with sarcopenia when combined with a physical activity intervention, such as resistance exercise.*

This statement received moderate agreement (73.4%) in Phase 2. Compelling free text responses were considered and incorporated to reflect expert opinion on the statement.

The evidence for combining adequate protein and energy intake with resistance exercise in persons with sarcopenia is low according to the ICFSR clinical practice guidelines.

**Statement 19**

**Optimisation of energy and protein intake is likely to be beneficial for all persons with sarcopenia, but benefits may be greatest when combined with a physical activity intervention, such as resistance exercise.**

**The following best represents my agreement with statement 19:**

| Strongly disagree | | | | Neither agree nor disagree | | | Strongly agree | | | |
| --- | --- | --- | --- | --- | --- | --- | --- | --- | --- | --- |
| 0 | 1 | 2 | 3 | 4 | 5 | 6 | 7 | 8 | 9 | 10 |
| I do not have an opinion on this statement | | | | | | | | | | |

Are there are any other comments you wish to make? _____________________________________

**Preamble for Statement 21**

In Phase 2, item 21 was a question requesting experts select which assessment elements they thought should be undertaken in an assessment of a patient with or suspected to have sarcopenia. While assessment for depression and anxiety was deemed least important by experts, this element was ranked highly by consumers. Therefore “mood assessment” has been included with “cognition assessment” in this statement preamble.

We have presented 10 items taking into account both consumer and expert feedback. We have termed these the “Basic Assessment Sarcopenia Items for Completion,” or “BASIC.” The BASIC items include;

1. **Sarcopenia diagnostic measures**
2. **Falls and fracture history**
3. **Functional status**
4. **Nutritional assessment**
5. **Physical activity levels**
6. **Quality of life and self-rated health**
7. **Medications history**
8. **Comorbidity assessment**
9. **Cognition and mood assessment**
10. **Social support assessment**

Further, consumers identified preferring a consultation length of either 30-60 minutes (37.5%) or “as long as it takes” (41.7%) in which these assessments would be undertaken. Therefore, this statement has been developed to reflect the preferences of consumers and experts.

**Statement 21**

**The ANZSSFR recommends clinicians undertake a consultation of 30-60 minutes duration with persons with or at risk of sarcopenia, which would include assessments described by the BASIC (Basic Assessment Sarcopenia Items for Completion).**

**The following best represents my agreement with statement 21:**

| Strongly disagree | | | | Neither agree nor disagree | | | Strongly agree | | | |
| --- | --- | --- | --- | --- | --- | --- | --- | --- | --- | --- |
| 0 | 1 | 2 | 3 | 4 | 5 | 6 | 7 | 8 | 9 | 10 |
| I do not have an opinion on this statement | | | | | | | | | | |

Are there any other comments you wish to make?

Thank you for completing this survey and your involvement in this important process. We will be in touch with the results in the coming weeks.
