# Supplementary material for: Consensus guidelines for sarcopenia prevention, diagnosis and management in Australia and New Zealand
Source: J Cachexia Sarcopenia Muscle. 2022 Nov 9;14(1):142–56. doi: 10.1002/jcsm.13115 (PMC9891980; doi:10.1002/jcsm.13115)
Supplement: Supplementary file 5 — Table S2. Population, Intervention, Comparison, Outcome (PICO) questions and GRADE assessments [file JCSM-14-142-s008.docx]

**Supplement 5 – Population, Intervention, Comparison, Outcome (PICO) questions and GRADE assessments**

| **State-ment** | **PICO question** | **Decision type** | **Key Reference(s)** | **Priority** | **Accuracy** | **Benefits (+) / harms (-)** | **Outcome importance** | **Balance favours** | **Resource use** | **Equity** | **Acceptability** | **Feasibility** | **Evidence strength summary** | **Evidence certainty*** | **Agreement (%)** | **Classification** |
| --- | --- | --- | --- | --- | --- | --- | --- | --- | --- | --- | --- | --- | --- | --- | --- | --- |
| 1 | For sarcopenia prevention, should lifestyle interventions be encouraged in adults and at what ages compared with usual care? | Clinical-recommendation – population | 1, 2 | High | N/A | +; moderate  -; minimal | High | Intervention | Favours intervention | Improved equity if access supported | Yes | Financial and resource limitations | Strong | Low | 100 | CBR |
| 2 | Should person-centred lifestyle interventions be offered in those with conditions likely to increase the risk of sarcopenia, compared with no intervention? | Clinical recommendation – individual | 1-3 | High | N/A | +; moderate to high  -; minimal | High | Intervention | Favours intervention | Improved equity if access supported | Yes (ref. *Consumer Delphi)* | Financial and resource limitations | Strong | Low | 93.6 | CBR |
| 3 | In people with sarcopenia, how frequently should assessments be undertaken compared with usual care? | Clinical recommendation – individual | 4 | Moderate | N/A | +; high -; minimal | Moderate | Intervention | Not answerable | Improved equity if access supported | Yes (ref. *Consumer Delphi)* | Financial and resource limitations | Weak | Low | 85.1 | CBR |
| 6 | In people with sarcopenia, is muscle mass important compared with other measures to determine sarcopenia? | Diagnostic, screening and other tests | 5 | Low | Test accuracy variable depending on method used | N/A | Low | N/A | Variable | Inequitable access to diagnostic services | Variable | Financial and resource limitations | Weak | Low | 90.9 | CBR |
| 7 | In older people with sarcopenia but in whom not all tests are possible, which tests make sarcopenia probable? | Diagnostic, screening and other tests | 6 | Moderate | Variable prevalence between testing methods | +; moderate  -; minimal | Moderate | N/A | Variable | Inequitable access to diagnostic services | Variable (ref. *Consumer Delphi)* | Financial and resource limitations | Moderate | Low | 80.0 | CBR |
| 8 | In different cultural and ethnic groups, what diagnostic cutpoints should be used to diagnose sarcopenia in comparison with standard measures? | Diagnostic, screening and other tests | 6, 7 | High | Variable prevalence between testing methods and populations | +; moderate  -; minimal | Moderate | N/A | Variable | Inequitable access to diagnostic services | Variable (ref. *Consumer Delphi)* | Financial and resource limitations | Moderate | Low | 86.2 | CBR |
| 13 | In persons with sarcopenia, should resistance-based exercise be offered by an accredited health professional as compared with no intervention? | Clinical recommendation – individual | 3 | High | N/A | +; high  -; minimal | High | Intervention | Favours intervention | Improved equity if access supported | Variable (ref. *Consumer Delphi)* | Financial and resource limitations | Strong | High | 92.5 | EBR |
| 14 | Should energy and protein intake be optimised in persons with sarcopenia, particularly in those undertaking a physical activity intervention such as resistance exercise? | Clinical recommendation – individual | 3 | Moderate | N/A | +; moderate  -; minimal | High | Intervention | Favours intervention | Improved equity if access supported | Variable (ref. *Consumer Delphi)* | Financial and resource limitations | Strong | Moderate | 97.9 | EBR |
| 15 | Should people with sarcopenia be referred to a dietitian for a calorie and protein optimisation plan, as compared to no referral? | Clinical recommendation – individual | 8 | Moderate | N/A | +; moderate  +; minimal | Moderate | Intervention | Equivocal | Improved equity if access supported | Variable (ref. *Consumer Delphi)* | Financial and resource limitations | Weak | Low | 90.9 | CBR |
| 16 | In people with sarcopenia, what is the optimal protein intake per kilogram compared with usual diet to optimise muscle strength and function? | Clinical recommendation – individual | 9 | Moderate | N/A | +; moderate  +; minimal | High | Intervention | Favours intervention | Improved equity if access supported | Variable (ref. *Consumer Delphi)* | Financial and resource limitations | Moderate | Low | 86.7 | CBR |

The GRADE EtD table was informed by references [35-36]. The search strategy comprised: i) the publication libraries of Task Force leads JZ and DS, and ii) PubMed database searches with search term combinations “sarcopenia” AND “prevention” OR” “screening” OR “diagnosis” OR “management” OR “treatment.” Statements outside the search strategy but approved via agreement ≥80% are classified as practice points (PP): 4,5, 9-12, 17.

PICO = Population, Intervention, Comparison, Outcome.

*Evidence certainty comprises imprecision, risk of bias, inconsistency, publication bias and indirectness, and the evidence underpinning each question is ranked as either 1; high – further research is very unlikely to change the confidence in the estimate of effect, 2; - moderate – further research is likely to have an important impact on confidence in the estimate of effect and may change the estimate, 3; low – further research is very likely to have an important impact on confidence in the estimate of effect and likely to change the estimate, 4; very low – any estimate of effect is very uncertain [34].

Table References
1. Granic A, Dismore L, Hurst C, Robinson SM, Sayer AA. Myoprotective Whole Foods, Muscle Health and Sarcopenia: A Systematic Review of Observational and Intervention Studies in Older Adults. Nutrients. 2020 Jul 28;12(8):2257. doi: 10.3390/nu12082257.

2. Steffl M, Bohannon RW, Sontakova L, Tufano JJ, Shiells K, Holmerova I. Relationship between sarcopenia and physical activity in older people: a systematic review and meta-analysis. Clin Interv Aging. 2017;12:835-845. doi:10.2147/CIA.S132940

3. Liao CD, Chen HC, Huang SW, Liou TH. The Role of Muscle Mass Gain Following Protein Supplementation Plus Exercise Therapy in Older Adults with Sarcopenia and Frailty Risks: A Systematic Review and Meta-Regression Analysis of Randomized Trials. Nutrients. 2019 Jul 25;11(8):1713. doi: 10.3390/nu11081713.

4. Dent E, Morley JE, Cruz-Jentoft AJ, Arai H, Kritchevsky SB, Guralnik J, Bauer JM, Pahor M, Clark BC, Cesari M, Ruiz J, Sieber CC, Aubertin-Leheudre M, Waters DL, Visvanathan R, Landi F, Villareal DT, Fielding R, Won CW, Theou O, Martin FC, Dong B, Woo J, Flicker L, Ferrucci L, Merchant RA, Cao L, Cederholm T, Ribeiro SML, Rodríguez-Mañas L, Anker SD, Lundy J, Gutiérrez Robledo LM, Bautmans I, Aprahamian I, Schols JMGA, Izquierdo M, Vellas B. International Clinical Practice Guidelines for Sarcopenia (ICFSR): Screening, Diagnosis and Management. J Nutr Health Aging. 2018;22(10):1148-1161. doi: 10.1007/s12603-018-1139-9.

5. Evans WJ, Hellerstein M, Orwoll E, Cummings S, Cawthon PM. D_3_ -Creatine dilution and the importance of accuracy in the assessment of skeletal muscle mass. J Cachexia Sarcopenia Muscle. 2019 Feb;10(1):14-21. doi: 10.1002/jcsm.12390.

6. Cruz-Jentoft AJ, Bahat G, Bauer J, Boirie Y, Bruyère O, Cederholm T, Cooper C, Landi F, Rolland Y, Sayer AA, Schneider SM, Sieber CC, Topinkova E, Vandewoude M, Visser M, Zamboni M; Writing Group for the European Working Group on Sarcopenia in Older People 2 (EWGSOP2), and the Extended Group for EWGSOP2. Sarcopenia: revised European consensus on definition and diagnosis. Age Ageing. 2019 Jan 1;48(1):16-31. doi: 10.1093/ageing/afy169.

7. Chen LK, Woo J, Assantachai P, Auyeung TW, Chou MY, Iijima K, Jang HC, Kang L, Kim M, Kim S, Kojima T, Kuzuya M, Lee JSW, Lee SY, Lee WJ, Lee Y, Liang CK, Lim JY, Lim WS, Peng LN, Sugimoto K, Tanaka T, Won CW, Yamada M, Zhang T, Akishita M, Arai H. Asian Working Group for Sarcopenia: 2019 Consensus Update on Sarcopenia Diagnosis and Treatment. J Am Med Dir Assoc. 2020 Mar;21(3):300-307.e2. doi: 10.1016/j.jamda.2019.12.012. Epub 2020 Feb 4.

8. Miller J, Wells L, Nwulu U, Currow D, Johnson MJ, Skipworth RJE. Validated screening tools for the assessment of cachexia, sarcopenia, and malnutrition: a systematic review. Am J Clin Nutr. 2018 Dec 1;108(6):1196-1208. doi: 10.1093/ajcn/nqy244.

9. Bauer J, Biolo G, Cederholm T, Cesari M, Cruz-Jentoft AJ, Morley JE, Phillips S, Sieber C, Stehle P, Teta D, Visvanathan R, Volpi E, Boirie Y. Evidence-based recommendations for optimal dietary protein intake in older people: a position paper from the PROT-AGE Study Group. J Am Med Dir Assoc. 2013 Aug;14(8):542-59.
