# Supplementary material for: Consensus guidelines for sarcopenia prevention, diagnosis and management in Australia and New Zealand
Source: J Cachexia Sarcopenia Muscle. 2022 Nov 9;14(1):142–56. doi: 10.1002/jcsm.13115 (PMC9891980; doi:10.1002/jcsm.13115)
Supplement: Supplementary file 6 — Data S4. Key literature [file JCSM-14-142-s010.docx]

**Supplement 6 – Key literature**

**Summaries and controversies**

1. Cruz-Jentoft AJ, Sayer AA. Sarcopenia. Lancet. 2019;393(10191):2636-46.
2. Cawthon PM. Recent Progress in Sarcopenia Research: a Focus on Operationalizing a Definition of Sarcopenia. Current osteoporosis reports. 2018;16(6):730-7.

**Definitions**

1. Baumgartner RN, Koehler KM, Gallagher D, Romero L, Heymsfield SB, Ross RR, et al. Epidemiology of sarcopenia among the elderly in New Mexico. American journal of epidemiology. 1998;147(8):755-63.
2. Cruz-Jentoft AJ, Bahat G, Bauer J, Boirie Y, Bruyere O, Cederholm T, et al. Sarcopenia: revised European consensus on definition and diagnosis. Age and ageing. 2019;48(1):16-31.
3. Cruz-Jentoft AJ, Baeyens JP, Bauer JM, Boirie Y, Cederholm T, Landi F, et al. Sarcopenia: European consensus on definition and diagnosis: Report of the European Working Group on Sarcopenia in Older People. Age and ageing. 2010;39(4):412-23.
4. Zanker J, Scott D, Reijnierse EM, Brennan-Olsen SL, Daly RM, Girgis CM, et al. Establishing an Operational Definition of Sarcopenia in Australia and New Zealand: Delphi Method Based Consensus Statement. The journal of nutrition, health & aging. 2019;23(1):105-10.
5. Chen LK, Liu LK, Woo J, Assantachai P, Auyeung TW, Bahyah KS, et al. Sarcopenia in Asia: consensus report of the Asian Working Group for Sarcopenia. Journal of the American Medical Directors Association. 2014;15(2):95-101.
6. Chen LK, Woo J, Assantachai P, Auyeung TW, Chou MY, Iijima K, et al. Asian Working Group for Sarcopenia: 2019 Consensus Update on Sarcopenia Diagnosis and Treatment. Journal of the American Medical Directors Association. 2020;21(3):300-7.e2.
7. Bhasin S, Travison TG, Manini TM, Patel S, Pencina KM, Fielding RA, et al. Sarcopenia Definition: The Position Statements of the Sarcopenia Definition and Outcomes Consortium. Journal of the American Geriatrics Society. 2020
8. Studenski SA, Peters KW, Alley DE, Cawthon PM, McLean RR, Harris TB, et al. The FNIH sarcopenia project: rationale, study description, conference recommendations, and final estimates. The journals of gerontology Series A, Biological sciences and medical sciences. 2014;69(5):547-58.

**Translation, knowledge and management**

1. Orwig D, Magaziner J, Fielding RA, Zhu H, Binder EF, Cawthon PM, et al. Application of SDOC Cut-points for Low Muscle Strength for Recovery of Walking Speed After Hip Fracture. The Journals of Gerontology: Series A. 2020.
2. Yeung SSY, Reijnierse EM, Trappenburg MC, Meskers CGM, Maier AB. Current knowledge and practice of Australian and New Zealand health-care professionals in sarcopenia diagnosis and treatment: Time to move forward! Australasian journal on ageing. 2019.
3. Dent E, Morley JE, Cruz-Jentoft AJ, Arai H, Kritchevsky SB, Guralnik J, et al. International Clinical Practice Guidelines for Sarcopenia (ICFSR): Screening, Diagnosis and Management. The journal of nutrition, health & aging. 2018;22(10):1148-61.
4. Liao CD, Chen HC, Huang SW, Liou TH. The Role of Muscle Mass Gain Following Protein Supplementation Plus Exercise Therapy in Older Adults with Sarcopenia and Frailty Risks: A Systematic Review and Meta-Regression Analysis of Randomized Trials. Nutrients. 2019 Jul 25;11(8):1713.
