# Supplementary material for: Consensus guidelines for sarcopenia prevention, diagnosis and management in Australia and New Zealand
Source: J Cachexia Sarcopenia Muscle. 2022 Nov 9;14(1):142–56. doi: 10.1002/jcsm.13115 (PMC9891980; doi:10.1002/jcsm.13115)
Supplement: Supplementary file 7 — Table S3. Classification of statements by agreement, and strength and certainty of evidence [file JCSM-14-142-s009.docx]

**Supplement 7 – Classification of statements by agreement, and strength and certainty of evidence**

| **Descriptor** | **Definition** | **Method of determination** |
| --- | --- | --- |
| Evidence-based recommendation (EBR) | Strength of evidence is “strong;”  AND   Certainty of evidence is high or moderate;  AND   Consensus achieved by survey respondents based on pre-specified level of agreement (>80%). | Strength of evidence regards the accuracy (for tests), benefit-harm balance, cost, patient preference and quality of evidence [33] “Strong” = benefits outweigh the risks.  AND  Certainty (quality) regards the certainty of evidence for effect [33] “High” – Further research unlikely to change confidence in the estimate of effect.  OR  “Moderate” – Further research is likely to have an importance impact on confidence in the estimate of effect and may change the estimate.  AND  Percentage of agreement with statement is at least 80% of respondents in at least moderate 7 or 8 out of 10) or high (9 or 10 out of 10) agreement. |
| Consensus-based recommendation (CBR) | “Formulated in the absence of quality evidence (where a systematic review of the evidence was conducted as part of the search strategy)” [32];  AND  Consensus achieved by survey respondents based on pre-specified level of agreement (>80%). | Criteria for EBR was not satisfied.  AND  Percentage of agreement with statement is at least 80% of respondents in at least moderate 7 or 8 out of 10) or high (9 or 10 out of 10) agreement. |
| Practice-point (PP) | “Recommendation(s) included in the guideline, where the subject matter is outside the scope of [the] search strategy” [32];  AND  Consensus achieved by survey respondents based on pre-specified level of agreement (>80%). | Criteria for EBR and CBR were not satisfied.  AND  Percentage of agreement with statement is at least 80% of respondents in at least moderate 7 or 8 out of 10) or high (9 or 10 out of 10) agreement. |

EBR = evidence-based recommendation; CBR = consensus-based recommendation; PP = practice-point.

Literature on grading the strength and certainty of evidence is available via the GRADE EtD framework [33–35]
