# Supplementary material for: Consensus guidelines for sarcopenia prevention, diagnosis and management in Australia and New Zealand
Source: J Cachexia Sarcopenia Muscle. 2022 Nov 9;14(1):142–56. doi: 10.1002/jcsm.13115 (PMC9891980; doi:10.1002/jcsm.13115)
Supplement: Supplementary file 8 — Figure S1. Results Phases 2 and 3 [file JCSM-14-142-s002.docx]

**Supplement 8 – Results Phases 2 and 3**

**Phase 2 Question 4 - The ANZSSFR should endorse which of the following screening tools for sarcopenia in those meeting the criteria in statement 3 (select all that apply)**

**
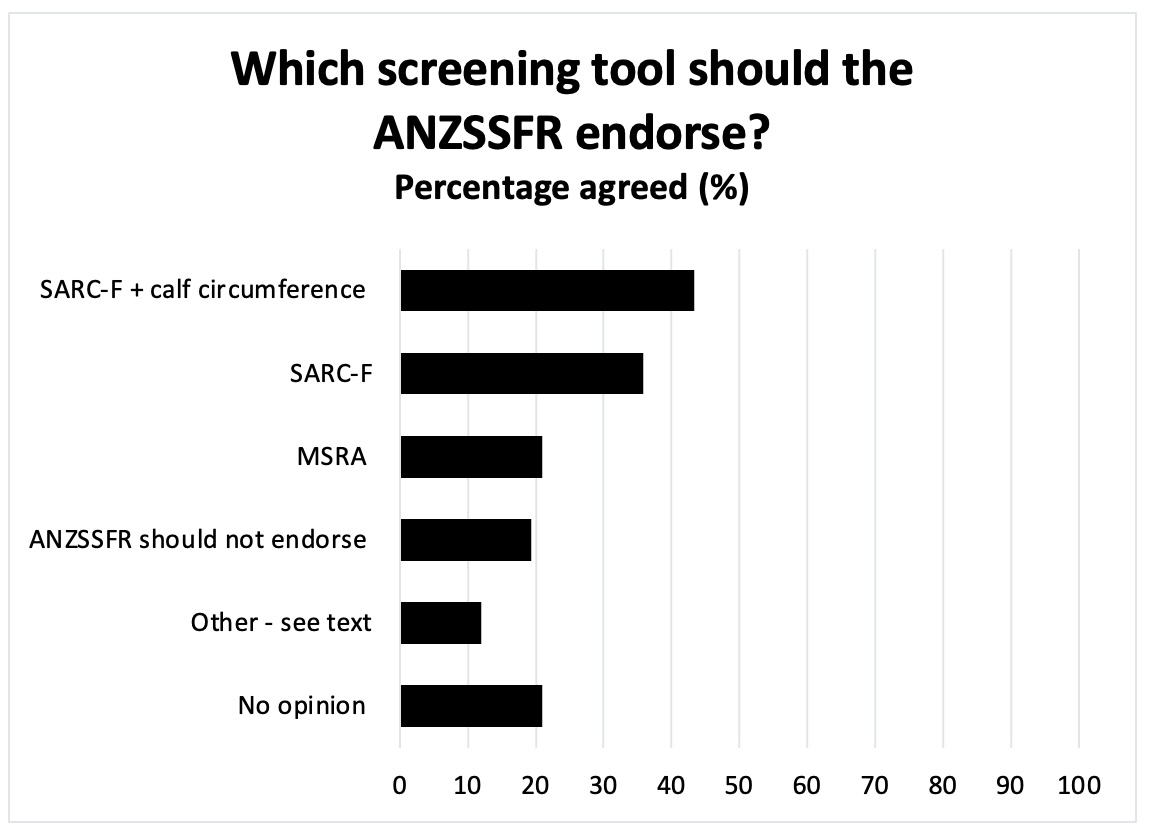
**

**Phase 2 Question 7 - My recommendation for measuring muscle strength, which considers clinical application, timing, equipment, and utility in predicting negative outcomes, is:**


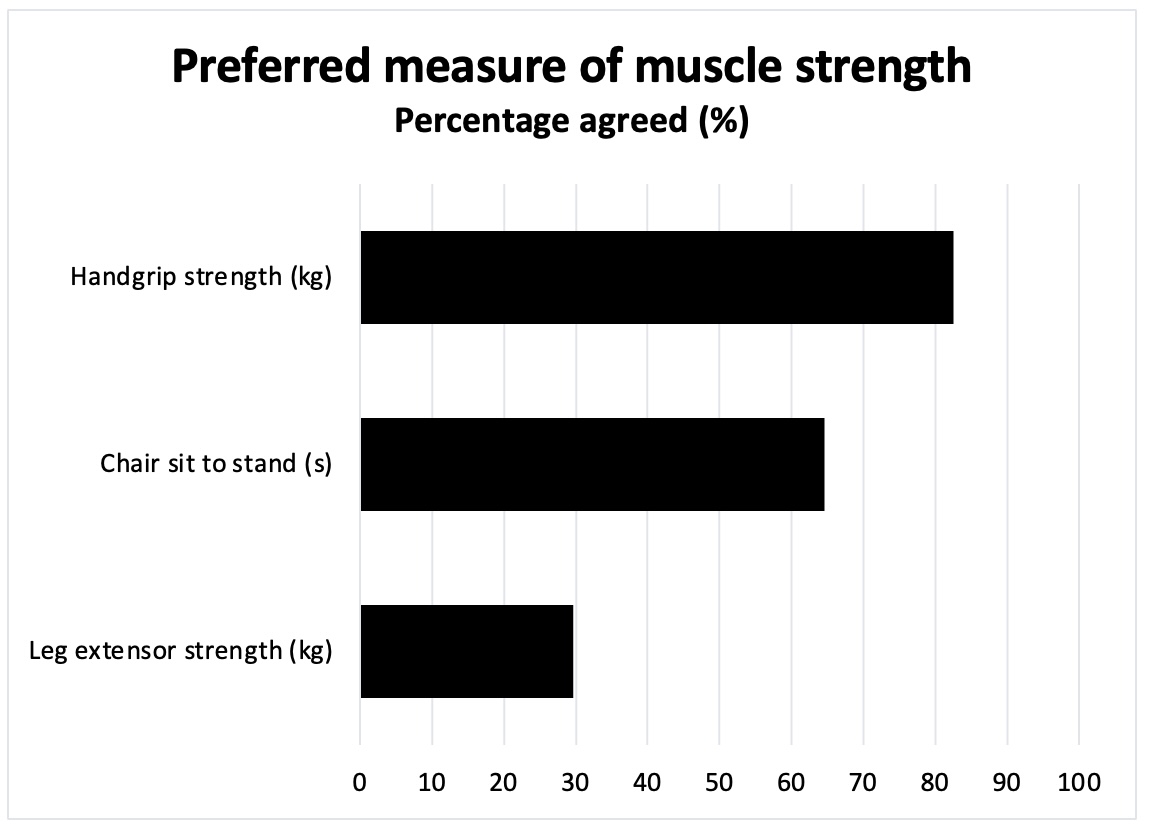


**Phase 2 Question 8 - My recommendation for measuring physical performance, which considers clinical application, timing, equipment, and utility in predicting negative outcomes, is:**

**
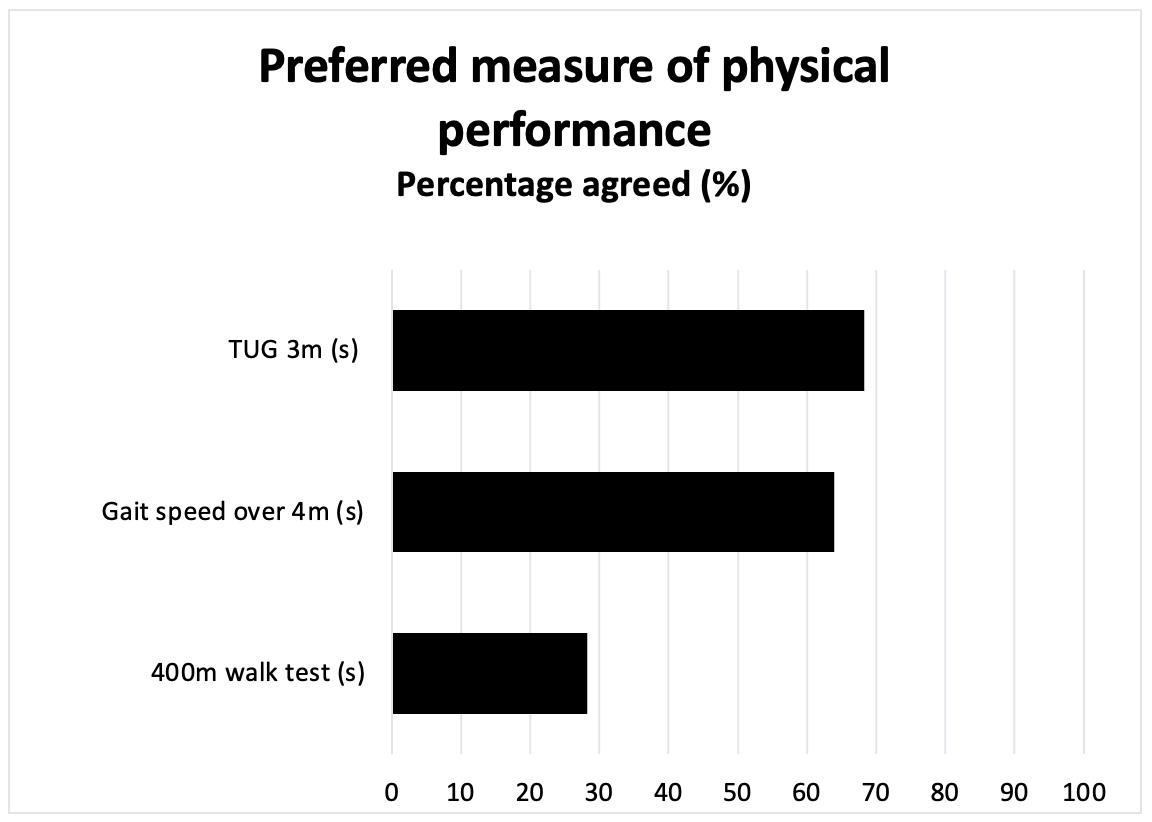
**

**Phase 2 Question 11 - The ANZSSFR should adopt the following operational definition and associated cut-points of sarcopenia for use by clinicians and researchers in Australia and New Zealand:**

**
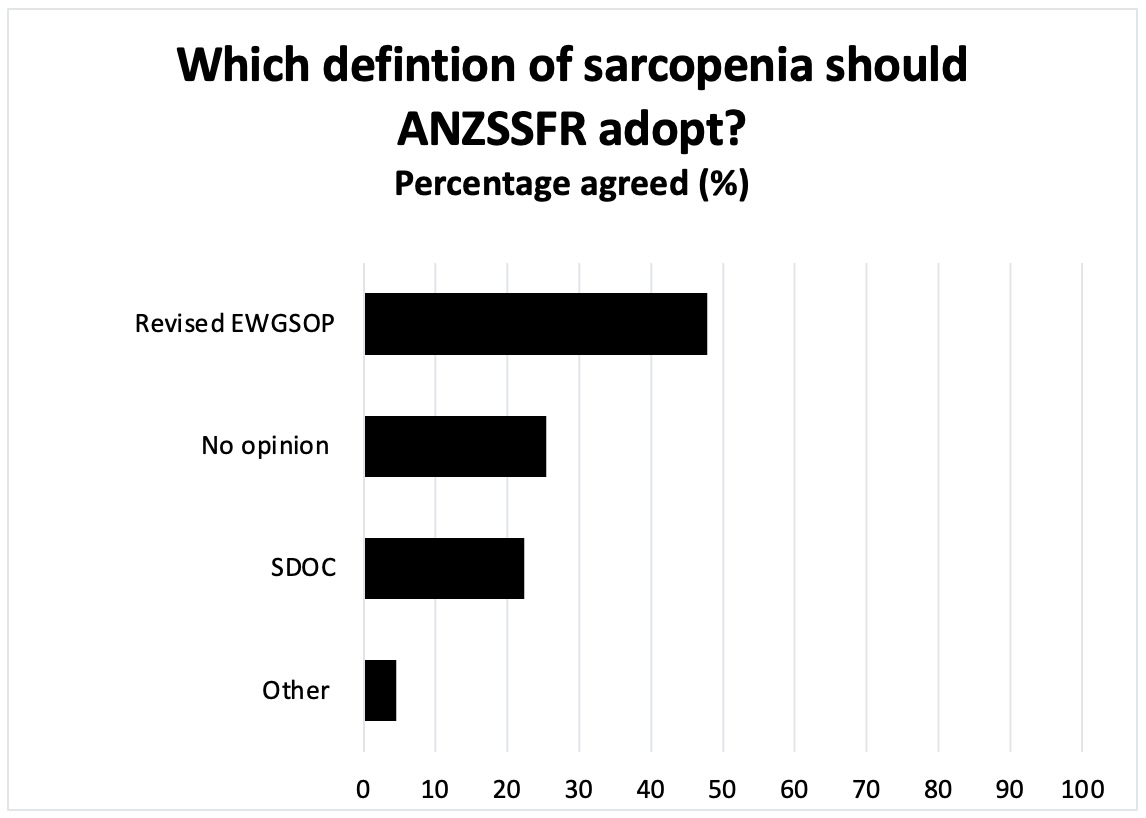
**

**Phase 2 Question 21 - Please select as many of the following as you deem appropriate in a consultation where a person is being assessed for sarcopenia, and feel free to add additional assessments in the free text below:**

**
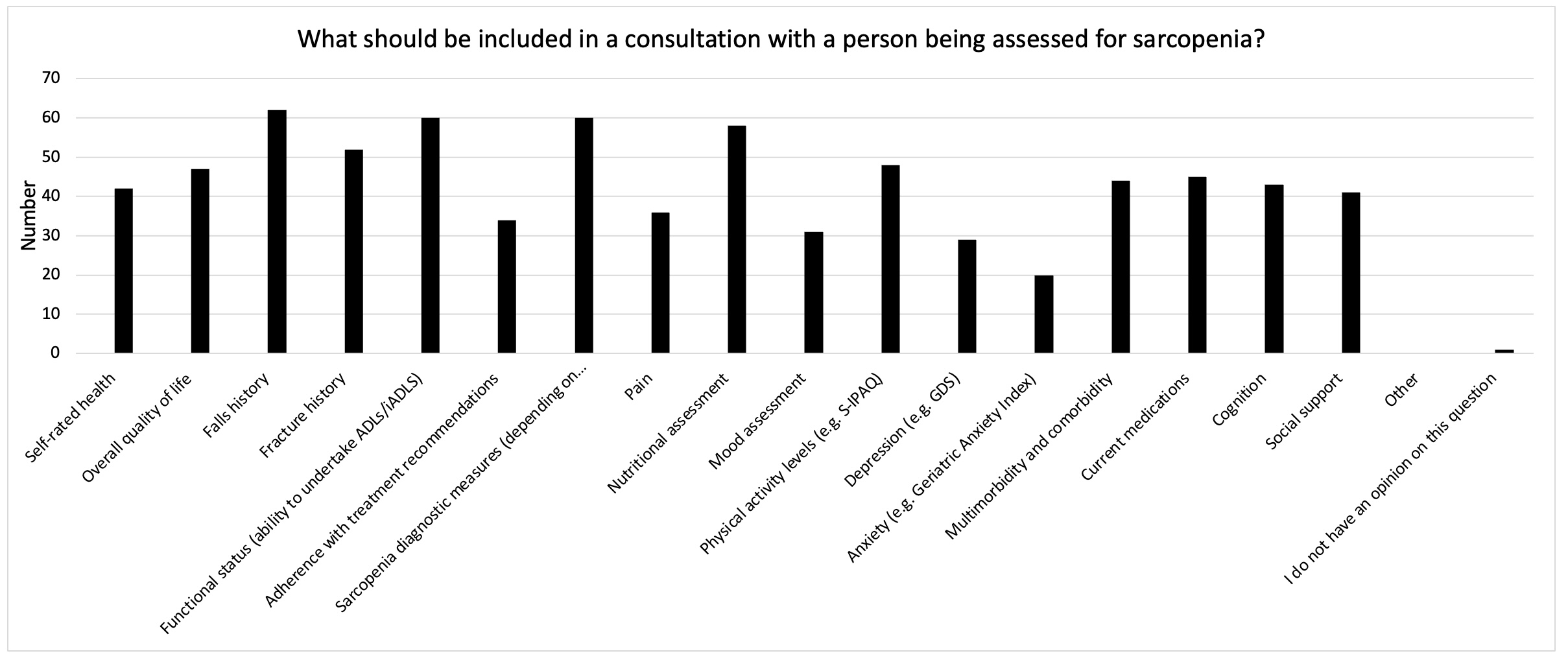
**

**Phase 3 Question 7 - Providing that access or uptake is not limited by patient-related factors or availability, the measure of muscle strength I recommend is:**


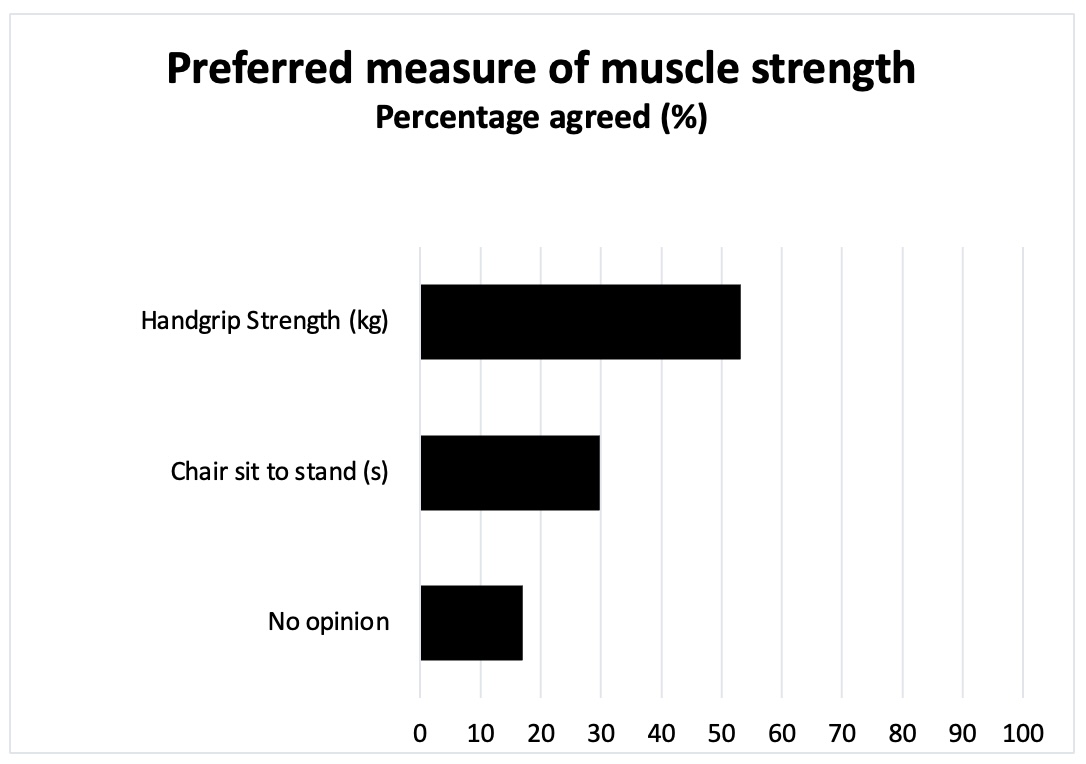


**Phase 3 Question 8 - Providing that access or uptake is not limited by patient-related factors or availability, the measure of physical performance I recommend is:**


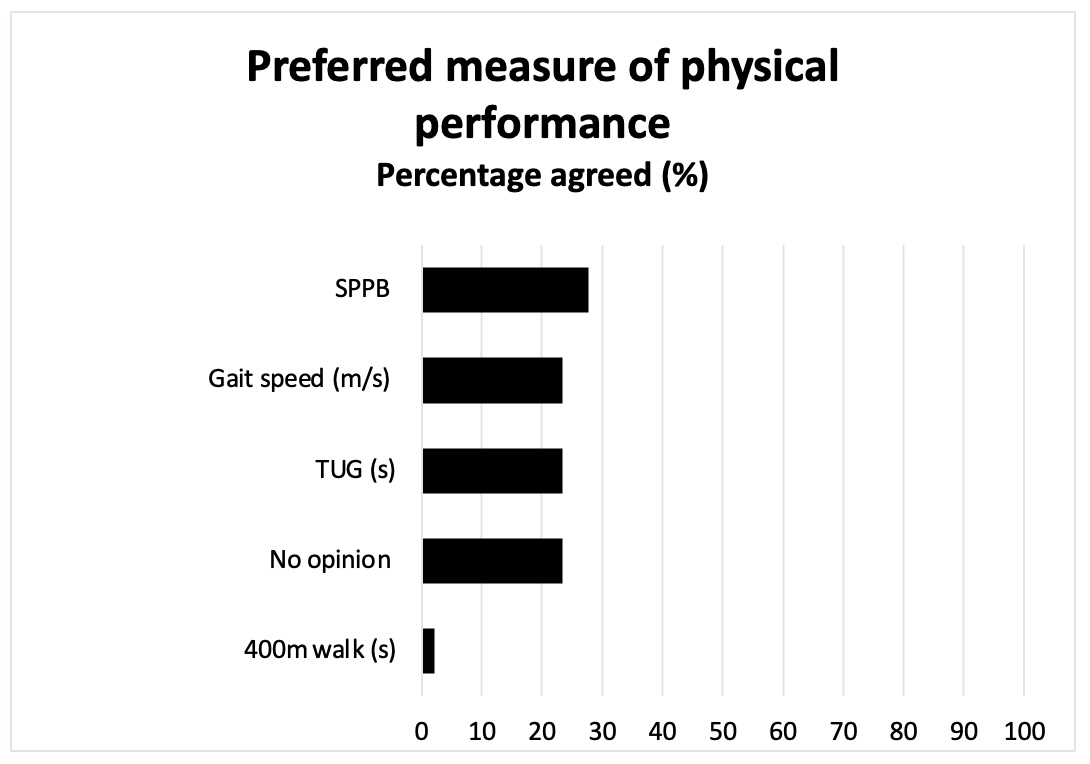


**Phase 3 Question 11 - Except where patient characteristics suggest the use of the revised Asian Working Group definition of sarcopenia is more appropriate, the definition of sarcopenia I recommend is:**

**
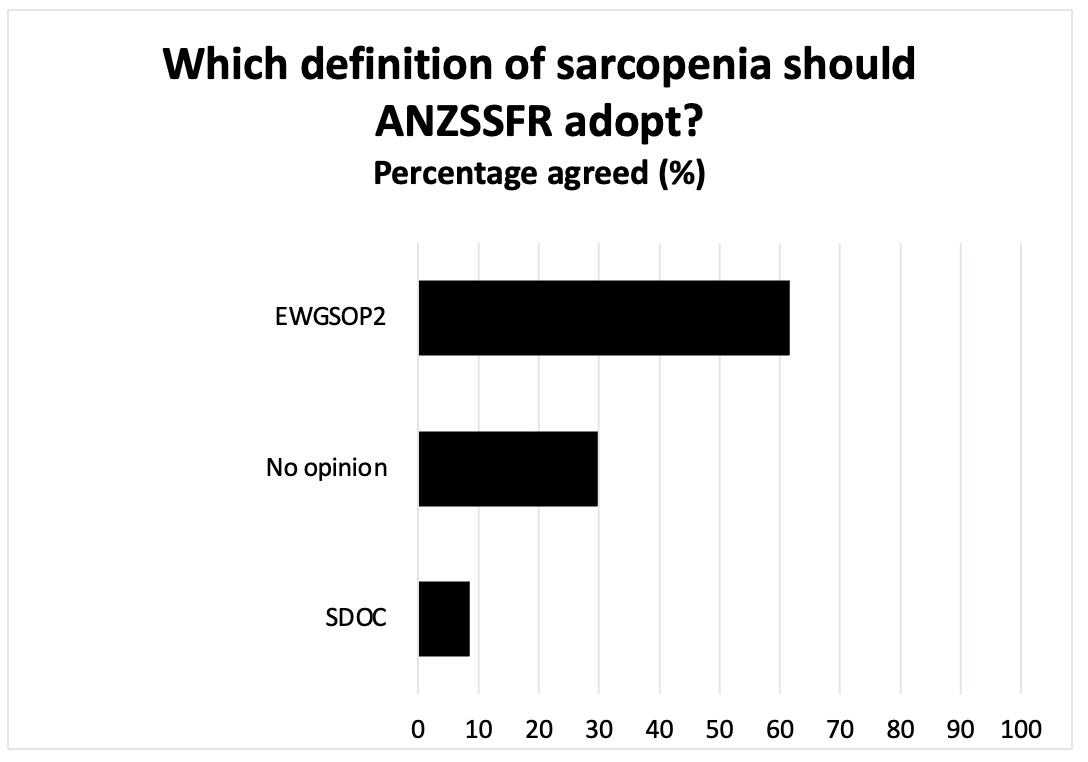
**

ANZSSFR = Australian and New Zealand Society for Sarcopenia and Frailty Research. EWGSOP2 = Revised European Working Group for Sarcopenia in Older Persons. . MSRA = Mini Sarcopenia Risk Assessment. SDOC = Sarcopenia Diagnostic and Outcomes Consortium. SPPB = Short Physical Performance Battery. TUG = Timed-Up-And-Go test over 3 metres.
