# Supplementary material for: Consensus guidelines for sarcopenia prevention, diagnosis and management in Australia and New Zealand
Source: J Cachexia Sarcopenia Muscle. 2022 Nov 9;14(1):142–56. doi: 10.1002/jcsm.13115 (PMC9891980; doi:10.1002/jcsm.13115)
Supplement: Supplementary file 9 — Data S5. The Basic Assessment Sarcopenia Items for Completion (BASIC) [file JCSM-14-142-s001.docx]

**Supplement 9 – The Basic Assessment Sarcopenia Items for Completion (BASIC)**

1. Sarcopenia diagnostic measures
2. Falls and fracture history
3. Functional status
4. Nutritional assessment
5. Physical activity levels
6. Quality of life and self-rated health
7. Medications history
8. Comorbidity assessment
9. Cognition and mood assessment
10. Social support assessment
