# Supplementary material for: Consensus guidelines for sarcopenia prevention, diagnosis and management in Australia and New Zealand
Source: J Cachexia Sarcopenia Muscle. 2022 Nov 9;14(1):142–56. doi: 10.1002/jcsm.13115 (PMC9891980; doi:10.1002/jcsm.13115)
Supplement: Supplementary file 10 — Data S6. References S1 to S22 [file JCSM-14-142-s003.docx]

**Supplement 10 – References S1 to S22**

S1. Liu JYJ, Reijnierse EM, van Ancum JM, Verlaan S, Meskers CGM, Maier AB. Acute inflammation is associated with lower muscle strength, muscle mass and functional dependency in male hospitalised older patients. PLoS ONE. 2019;14(4):e215097

S2. Aarden JJ, Reijnierse EM, van der Schaaf M, van der Esch M, Reichardt LA, van Seben R, et al. Longitudinal Changes in Muscle Mass, Muscle Strength, and Physical Performance in Acutely Hospitalized Older Adults. J Am Med Dir Assoc. 2021;22(4):839-845.e1.

S3. Daly RM, Iuliano S, Fyfe JJ, Scott D, Kirk B, Thompson MQ, et al. Screening, Diagnosis and Management of Sarcopenia and Frailty in Hospitalized Older Adults: Recommendations from the Australian and New Zealand Society for Sarcopenia and Frailty Research (ANZSSFR) Expert Working Group. J Nutr Health Aging. 2022;26:637-651.

S4. Dodds RM, Syddall HE, Cooper R, Kuh D, Cooper C, Avan Aihie Sayer. Global variation in grip strength: a systematic review and meta-analysis of normative data. Age Ageing. 2016;45(2):209–16.

S5. Oh SS, Galanter J, Thakur N, Pino-Yanes M, Barcelo NE, White MJ, et al. Diversity in Clinical and Biomedical Research: A Promise Yet to Be Fulfilled. PLoS Med. 2015;12(12):e1001918.

S6. Yeung SSY, Reijnierse EM, Trappenburg MC, Meskers CGM, Maier AB. Current knowledge and practice of Australian and New Zealand health-care professionals in sarcopenia diagnosis and treatment: Time to move forward! Australas J Ageing. 2020;39(2):e185-e193.

S7. Malmstrom TK, Miller DK, Simonsick EM, Ferrucci L, Morley JE. SARC‐F: a symptom score to predict persons with sarcopenia at risk for poor functional outcomes. J Cachexia Sarcopenia Muscle. 2016;7(1):28–36.

S8. Voelker SN, Michalopoulos N, Maier AB, Reijnierse EM. Reliability and Concurrent Validity of the SARC-F and Its Modified Versions: A Systematic Review and Meta-Analysis. J Am Med Dir Assoc. 2021;22(9):1864-1876.e16.

S9. Dedeyne L, Reijnierse EM, Pacifico J, Kay JE, Maggs P, Verschueren S, et al. SARC-F Is Inaccurate to Identify Geriatric Rehabilitation Inpatients at Risk for Sarcopenia: RESORT. Gerontol. 2022;68:252-260.

S10. Cesari M, Marzetti E, Calvani R. Sarcopenia and SARC-F: “Perfect is the Enemy of Good.” J Am Med Dir Assoc. 2021;22(9):1862–3.

S11. Evans WJ, Hellerstein M, Orwoll E, Cummings S, Cawthon PM. D3 -Creatine dilution and the importance of accuracy in the assessment of skeletal muscle mass. J Cachexia Sarcopenia Muscle. 2019;10(1):14-21

S12. Zanker J, Patel S, Blackwell T, Duchowny K, Brennan-Olsen S, Cummings SR, et al. Walking Speed and Muscle Mass Estimated by the D3-Creatine Dilution Method Are Important Components of Sarcopenia Associated With Incident Mobility Disability in Older Men: A Classification and Regression Tree Analysis. J Am Med Dir Assoc. 2020;21(12):1997-2002.e1.

S13. Zanker J, Blackwell T, Patel S, Duchowny K, Brennan-Olsen S, Cummings SR, et al. Factor analysis to determine relative contributions of strength, physical performance, body composition and muscle mass to disability and mobility disability outcomes in older men. Exp Gerontol. 2022;161:111714.

S14. Orwig DL, Magaziner J, Fielding RA, Zhu H, Binder EF, Cawthon PM, et al. Application of SDOC Cut Points for Low Muscle Strength for Recovery of Walking Speed After Hip Fracture. J Gerontol A Biol Sci Med Sci. 2020;75(7):1379–85.

S15. Perera S, Mody SH, Woodman RC, Studenski SA. Meaningful Change and Responsiveness in Common Physical Performance Measures in Older Adults. J Am Geriatr Soc. 2006;54(5):743–9.

S16. Tieland M, Verdijk LB, de Groot LCPGM, van Loon LJC. Handgrip strength does not represent an appropriate measure to evaluate changes in muscle strength during an exercise intervention program in frail older people. Int J Sport Nutr Exerc Metab. 2015;25(1):27–36.

S17. Bombard Y, Baker GR, Orlando E, Fancott C, Bhatia P, Casalino S, et al. Engaging patients to improve quality of care: A systematic review. Implementation Science. 2018;13(1):1–22.

S18. Izquierdo M, Merchant RA, Morley JE, Anker SD, Aprahamian I, Arai H, et al. International Exercise Recommendations in Older Adults (ICFSR): Expert Consensus Guidelines. J Nutr Health Aging. 2021;25(7):824–53.

S19. Liao C de, Chen HC, Huang SW, Liou TH. The Role of Muscle Mass Gain Following Protein Supplementation Plus Exercise Therapy in Older Adults with Sarcopenia and Frailty Risks: A Systematic Review and Meta-Regression Analysis of Randomized Trials. Nutri. 2019;11(8):1713.

S20. Oktaviana J, Zanker J, Vogrin S, Duque G. The effect of protein supplements on functional frailty in older persons: A systematic review and meta-analysis. Arch Gerontol Geriatr. 2019;86:103938–103938.

S21. Bauer J, Biolo G, Cederholm T, Cesari M, Cruz-Jentoft AJ, Morley JE, et al. Evidence-based recommendations for optimal dietary protein intake in older people: a position paper from the PROT-AGE Study Group. J Am Med Dir Assoc. 2013;14(8):542–59.

S22. Venus C, Jamrozik E. Transparency in clinical practice guidelines: the problem of consensus-based recommendations and practice points. Intern Med J. 2021;51(2):291–4.
